# Supplementary material for: Prevalence of stroke in young adults in the Middle East and North Africa Region: A systematic review and meta-analysis
Source: PLOS Glob Public Health. 2025 Oct 6;5(10):e0004666. doi: 10.1371/journal.pgph.0004666 (PMC12500109; doi:10.1371/journal.pgph.0004666)
Supplement: S1 Text — (DOCX) [file pgph.0004666.s001.docx]

**S1 Text:** **Search Strategy**

**Date of search: 20th January 2024**

Total: 5939

- PubMed: 1527
- Embase: 2,366
- Scopus: 1,630
- CINHAL: 416

Duplicates removed on EndNote: 2656

Remaining 3283

Duplicates removed on Rayyan:

**Date of update: 29^th^ November 2024**

Total 392

- Pubmed 112
- Embase 132
- Scopus 123
- CINHAL 25

Duplicates removed on EndNote: 179

Duplicates removed on Rayyan: 22

Remaining 191

**PubMed:**

("Stroke"[Mesh] OR "Stroke, Lacunar"[Mesh] OR "Hemorrhagic Stroke"[Mesh] OR "Embolic Stroke"[Mesh] OR "Thrombotic Stroke"[Mesh] OR "Ischemic Stroke"[Mesh] OR "Brain Stem Infarctions"[Mesh] OR stroke* [title] OR “brain infarction” [tiab] OR "lacunar stroke*" [tiab] OR "hemorrhagic stroke*" [tiab] OR "embolic stroke*" [tiab] OR "thrombotic stroke*" [tiab] OR "ischemic stroke*" [tiab] OR "brain stem infarction*" [tiab] OR “cerebrovascular accident*” [tiab] OR “cerebrovascular event*” [tiab] OR “cerebrovascular disease*” [tiab] OR “cerebral infarction*” [tiab] OR CVA [title])

AND (“MENA” [tiab] OR “Middle East*” [tiab] OR “North Africa*” [tiab] OR “Algeria*” [tiab] OR Bahrain* [tiab] OR Djibouti* [tiab] OR Egypt* [tiab] OR Iran* [tiab] OR Iraq* [tiab] OR Jordan* [tiab] OR Kuwait* [tiab] OR Leban* [tiab] OR Liby* [tiab] OR Malt* [tiab] OR Mauritania* [tiab] OR Morocc* [tiab] OR Oman* [tiab] OR Palestin* [tiab] OR Qatar* [tiab] OR “Saudi” [tiab] OR Somalia* [tiab] OR Sudan* [tiab] OR Syria* [tiab] OR Tunisia* [tiab] OR “United Arab Emirates” [tiab] OR UAE [title] OR Emirati [tiab] OR Yemen* [tiab])

**Embase:**

(stroke*:ti OR 'brain infarction':ti,ab OR 'lacunar stroke*':ti,ab OR 'hemorrhagic stroke*':ti,ab OR 'embolic stroke*':ti,ab OR 'thrombotic stroke*':ti,ab OR 'ischemic stroke*':ti,ab OR 'brain stem infarction*':ti,ab OR 'cerebrovascular accident*':ti,ab OR 'cerebrovascular event*':ti,ab OR 'cerebrovascular disease*':ti,ab OR 'cerebral infarction*':ti,ab OR CVA:ti)

AND (MENA:ti,ab OR 'Middle East*':ti,ab OR 'North Africa*':ti,ab OR Algeria*:ti,ab OR Bahrain*:ti,ab OR Djibouti*:ti,ab OR Egypt*:ti,ab OR Iran*:ti,ab OR Iraq*:ti,ab OR Jordan*:ti,ab OR Kuwait*:ti,ab OR Leban*:ti,ab OR Liby*:ti,ab OR Malt*:ti,ab OR Mauritania*:ti,ab OR Morocc*:ti,ab OR Oman*:ti,ab OR Palestin*:ti,ab OR Qatar*:ti,ab OR Saudi:ti,ab OR Somalia*:ti,ab OR Sudan*:ti,ab OR Syria*:ti,ab OR Tunisia*:ti,ab OR 'United Arab Emirates':ti,ab OR UAE:ti OR Emirati:ti,ab OR Yemen*:ti,ab)

**Scopus:**

(TITLE(stroke*) OR TITLE-ABS("brain infarction") OR TITLE-ABS("lacunar stroke*") OR TITLE-ABS("hemorrhagic stroke*") OR TITLE-ABS("embolic stroke*") OR TITLE-ABS("thrombotic stroke*") OR TITLE-ABS("ischemic stroke*") OR TITLE-ABS("brain stem infarction*") OR TITLE-ABS("cerebrovascular accident*") OR TITLE-ABS("cerebrovascular event*") OR TITLE-ABS("cerebrovascular disease*") OR TITLE-ABS("cerebral infarction*") OR TITLE(CVA))

AND (TITLE-ABS(MENA) OR TITLE-ABS("Middle East*") OR TITLE-ABS("North Africa*") OR TITLE-ABS(Algeria*) OR TITLE-ABS(Bahrain*) OR TITLE-ABS(Djibouti*) OR TITLE-ABS(Egypt*) OR TITLE-ABS(Iran*) OR TITLE-ABS(Iraq*) OR TITLE-ABS(Jordan*) OR TITLE-ABS(Kuwait*) OR TITLE-ABS(Leban*) OR TITLE-ABS(Liby*) OR TITLE-ABS(Malt*) OR TITLE-ABS(Mauritania*) OR TITLE-ABS(Morocc*) OR TITLE-ABS(Oman*) OR TITLE-ABS(Palestin*) OR TITLE-ABS(Qatar*) OR TITLE-ABS(Saudi) OR TITLE-ABS(Somalia*) OR TITLE-ABS(Sudan*) OR TITLE-ABS(Syria*) OR TITLE-ABS(Tunisia*) OR TITLE-ABS("United Arab Emirates") OR TITLE(UAE) OR TITLE-ABS(Emirati) OR TITLE-ABS(Yemen*))

**CINHAL:**

((TI stroke*) OR (TI "brain infarction" OR AB "brain infarction") OR (TI "lacunar stroke*" OR AB "lacunar stroke*") OR (TI "hemorrhagic stroke*" OR AB "hemorrhagic stroke*") OR (TI "embolic stroke*" OR AB "embolic stroke*") OR (TI "thrombotic stroke*" OR AB "thrombotic stroke*") OR (TI "ischemic stroke*" OR AB "ischemic stroke*") OR (TI "brain stem infarction*" OR AB "brain stem infarction*") OR (TI "cerebrovascular accident*" OR AB "cerebrovascular accident*") OR (TI "cerebrovascular event*" OR AB "cerebrovascular event*") OR (TI "cerebrovascular disease*" OR AB "cerebrovascular disease*") OR (TI "cerebral infarction*" OR AB "cerebral infarction*") OR (TI CVA))

AND ((TI MENA OR AB MENA) OR (TI "Middle East*" OR AB "Middle East*") OR (TI "North Africa*" OR AB "North Africa*") OR (TI Algeria* OR AB Algeria*) OR (TI Bahrain* OR AB Bahrain*) OR (TI Djibouti* OR AB Djibouti*) OR (TI Egypt* OR AB Egypt*) OR (TI Iran* OR AB Iran*) OR (TI Iraq* OR AB Iraq*) OR (TI Jordan* OR AB Jordan*) OR (TI Kuwait* OR AB Kuwait*) OR (TI Leban* OR AB Leban*) OR (TI Liby* OR AB Liby*) OR (TI Malt* OR AB Malt*) OR (TI Mauritania* OR AB Mauritania*) OR (TI Morocc* OR AB Morocc*) OR (TI Oman* OR AB Oman*) OR (TI Palestin* OR AB Palestin*) OR (TI Qatar* OR AB Qatar*) OR (TI Saudi OR AB Saudi) OR (TI Somalia* OR AB Somalia*) OR (TI Sudan* OR AB Sudan*) OR (TI Syria* OR AB Syria*) OR (TI Tunisia* OR AB Tunisia*) OR (TI "United Arab Emirates" OR AB "United Arab Emirates") OR (TI UAE) OR (TI Emirati OR AB Emirati) OR (TI Yemen* OR AB Yemen*))
