# Supplementary material for: Prevalence of stroke in young adults in the Middle East and North Africa Region: A systematic review and meta-analysis
Source: PLOS Glob Public Health. 2025 Oct 6;5(10):e0004666. doi: 10.1371/journal.pgph.0004666 (PMC12500109; doi:10.1371/journal.pgph.0004666)
Supplement: S1 Table — (DOCX) [file pgph.0004666.s003.docx]

**S1 Table:** Risk of Bias Assessment - Detailed Hoy Scale Scores for Each Included Study (Adapted from Hoy et al. 2012 [1])

| **Reference** | **Study citation** | **Was the study’s target population a close representation of the national population in relation to relevant variables, e.g. age, sex, occupation?** | **Was the sampling frame a true or close representation of the target population?** | **Was some form of random selection used to select the sample, OR, was a census undertaken?** | **Was the likelihood of non-response bias minimal?** | **Were data collected directly from the subjects (as opposed to a proxy)?** | **Was an acceptable case definition used in the study?** | **Was the study instrument that measured the parameter of interest (e.g. prevalence of low back pain) shown to have reliability and validity (if necessary)?** | **Was the same mode of data collection used for all subjects?** | **Was the length of the shortest prevalence period for the parameter of interest appropriate?** | **Were the numerator( s) and denominato r(s) for the parameter of interest appropriate?** | **Overall Risk of bias** |
| --- | --- | --- | --- | --- | --- | --- | --- | --- | --- | --- | --- | --- |
| [28] | Azarpazhooh et al., 2013 | 0 | 0 | 0 | 0 | 1 | 1 | 1 | 1 | 1 | 1 | 6 |
| [29] | Abujaber et al., 2024 | 1 | 0 | 0 | 0 | 0 | 1 | 1 | 0 | 1 | 0 | 4 |
| [30] | Alhazzani et al., 2018 | 1 | 0 | 0 | 0 | 0 | 1 | 1 | 0 | 1 | 0 | 4 |
| [31] | Chraa et al., 2014 | 1 | 0 | 0 | 0 | 0 | 1 | 1 | 0 | 1 | 0 | 4 |
| [32] | Khedr et al., 2014 | 0 | 1 | 1 | 1 | 1 | 1 | 0 | 1 | 1 | 1 | 8 |
| [33] | Lahoud et al., 2016 | 0 | 1 | 1 | 1 | 1 | 1 | 0 | 1 | 1 | 1 | 8 |
| [34] | Al-Rubeaan et al., 2016 | 0 | 0 | 1 | 1 | 1 | 1 | 0 | 1 | 1 | 1 | 7 |
| [35] | Amiri et al., 2018 | 0 | 1 | 1 | 1 | 1 | 1 | 0 | 1 | 1 | 1 | 8 |
| [36] | El Tallawy et al., 2015 | 0 | 1 | 1 | 1 | 1 | 1 | 0 | 1 | 1 | 1 | 8 |
| [37] | El Tallawy et al., 2013 | 0 | 1 | 1 | 1 | 1 | 1 | 0 | 1 | 1 | 1 | 8 |
| [38] | Engels et al., 2014 | 0 | 1 | 1 | 1 | 1 | 1 | 0 | 1 | 1 | 1 | 8 |
| [39] | Farghaly et al., 2013 | 0 | 1 | 1 | 1 | 1 | 1 | 0 | 1 | 1 | 1 | 8 |
| [12] | Ghandehari et al., 2006 | 0 | 1 | 1 | 1 | 1 | 1 | 1 | 1 | 1 | 0 | 8 |
| [40] | Khan et al., 2008 | 1 | 0 | 0 | 0 | 0 | 1 | 1 | 0 | 1 | 0 | 4 |

**References**

1. Hoy, D., et al., *Assessing risk of bias in prevalence studies: modification of an existing tool and evidence of interrater agreement.* Journal of Clinical Epidemiology, 2012. **65**(9): p. 934-939.
