# Supplementary material for: Prevalence of stroke in young adults in the Middle East and North Africa Region: A systematic review and meta-analysis
Source: PLOS Glob Public Health. 2025 Oct 6;5(10):e0004666. doi: 10.1371/journal.pgph.0004666 (PMC12500109; doi:10.1371/journal.pgph.0004666)
Supplement: S2 Table — (DOCX) [file pgph.0004666.s004.docx]

**S2 Table:** Extracted Data from the included studies

| citation | country | region | design | studydesign | type | T<45 | TS<45 | TM<45 | TSM<45 | TF<45 | TSF<45 | TI<45 | TH<45 | HOY | study |
| --- | --- | --- | --- | --- | --- | --- | --- | --- | --- | --- | --- | --- | --- | --- | --- |
| Azarpazhooh et al., 2013 (a) | Iran | Middle East | Population-based | population-based prospective cohort study | 0 | 24021 | 2 | 9639 | 0 | 14382 | 2 |  |  | 6 | Incidence of first ever stroke during Hajj ceremony |
| Azarpazhooh et al., 2013 (b) | Iran | Middle East | Population-based | MSIS | 0 | 253462 | 3 | 126926 | 1 | 126536 | 2 |  |  | 6 | Incidence of first ever stroke during Hajj ceremony |
| Abujaber et al, 2024 | Qatar | Middle East | Hospital-based | Hospital-based prospective observational study | 1 |  | 1746 |  |  |  |  | 1389 | 375 | 4 | Stroke in Qatar: a decade of insights from a national registry |
| Alhazzani et al., 2018 | Saudi Arabia | Middle East | Hospital-based | Prospective observational study | 0 | 1710560 | 187 | 920598 | 110 | 770295 | 77 |  |  | 4 | Study of stroke incidence in aseer region, Southwestern Saudi Arabia |
| Chraa et al., 2014 | Morocco | North Africa | Hospital-based | Retrospective study | 0 | 1000000 | 128 |  | 76 |  | 76 | 128 | 0 | 4 | Stroke in young adults: about 128 cases |
| Khedr et al., 2014 | Egypt | North Africa | Population-based | Cross sectional community-based study | 1 | 6018 | 4 | 3171 | 2 | 2847 | 2 | 4 | 0 | 8 | Prevalence of ischemic and hemorrhagic strokes in Qena governorate, Egypt: Community-based study |
| Lahoud et al., 2016 | Lebanon | Middle East | Population-based | Cross sectional community-based study | 0 | 5213 | 3 | 2593 | 0 | 2620 | 3 |  |  | 8 | Prevalence of Lebanese stroke survivors: A comparative pilot study |
| Al-Rubeaan et al., 2016 | Saudi Arabia | Middle East | Hospital-based | Retrospective Cross-sectional study | 0 | 11197 | 57 | 4687 | 38 | 6510 | 19 | 57 | 0 | 7 | Ischemic Stroke and Its Risk Factors in a Registry-Based Large Cross-Sectional Diabetic Cohort in a Country Facing a Diabetes Epidemic |
| Amiri et al., 2018 | Iran | Middle East | Population-based | population-based cohort study | 0 | 369343 | 58 | 186087 | 26 | 183256 | 32 |  |  | 8 | The Incidence and Characteristics of Stroke in Urban-Dwelling Iranian Women |
| El Tallawy et al., 2015 | Egypt | North Africa | Population-based | population based survey | 1 | 31998 | 21 | 15760 | 8 | 16238 | 13 | 17 | 4 | 8 | Epidemiology and clinical presentation of stroke in upper egypt (Desert area) |
| El Tallawy et al., 2013 | Egypt | North Africa | Population-based | population based survey | 0 | 11664 | 3 |  |  |  |  |  |  | 8 | Epidemiology of non-fatal cerebrovascular stroke and transient ischemic attacks in Al Quseir, Egypt |
| Engels et al., 2014 | Morocco | North Africa | Population-based | population-based survey | 0 | 30740 | 8 |  |  |  |  |  |  | 8 | Socioeconomic status and stroke prevalence in morocco:results from the rabat-casablanca study |
| Farghaly et al., 2013 | Egypt | North Africa | Population-based | population-based survey | 1 | 47101 | 25 |  |  |  |  | 19 | 6 | 8 | Epidemiology of nonfatal stroke and transient ischemic attack in Al-Kharga District, New Valley, Egypt |
| Ghandehari et al., 2006 | Iran | Middle East | Population-based | Incidence population-based study | 0 | 314000 | 124 | 158256 | 64 | 155744 | 60 | 124 | 0 | 8 | Incidence and etiology of ischemic stroke in Persian young adults |
| Khan et al., 2008 | Qatar | Middle East | Hospital-based | Hospital-based prospective observational study | 1 |  | 55 |  | 43 |  | 12 | 40 | 15 | 4 | Stroke in Qatar: A First Prospective Hospital-based Study of Acute Stroke |

**Names of data extractors:** Salma Hegazi, Roaa Aly, Duaa Yousif, Salma Al-Nuaimi

**Last date of data extraction:** November 2024
