# Supplementary material for: Prevalence of stroke in young adults in the Middle East and North Africa Region: A systematic review and meta-analysis
Source: PLOS Glob Public Health. 2025 Oct 6;5(10):e0004666. doi: 10.1371/journal.pgph.0004666 (PMC12500109; doi:10.1371/journal.pgph.0004666)
Supplement: S3 Table — (DOCX) [file pgph.0004666.s005.docx]

**S3 Table:** Studies Excluded from the Systematic Review and Reasons for Exclusion

| **title** | **ear** | **journal** | **issn** | **volume** | **issue** | **pages** | **authors** | **url** | **language** | **location** | **doi** | **Reason of Exclusion** | **Reviewers' Names** |
| --- | --- | --- | --- | --- | --- | --- | --- | --- | --- | --- | --- | --- | --- |
| Stroke burden in Egypt: Data from five epidemiological studies | 2019 | Journal of the Neurological Sciences | 1878-5883 0022-510X | 405 |  | 10-Sep | Abd-Allah, F. and Khedr, E. and Oraby, M. and Reda, R. | https://www.embase.com/search/results?subaction=viewrecord&id=L2004005855&from=export http://dx.doi.org/10.1016/j.jns.2019.10.775 | English |  | 10.1016/j.jns.2019.10.775 | conference abstract | Salma, Roaa |
| Burden of stroke in Egypt: Current status and opportunities | 2014 | International Journal of Stroke | | 9 | 8 | 1105-1108 | Abd-Allah, F. and Moustafa, R. R. | https://www.scopus.com/inward/record.uri?eid=2-s2.0-84920392413&doi=10.1111%2fijs.12313&partnerID=40&md5=72ed18114ad47f1e0e0b9d1ce4778989 | | | 10.1111/ijs.12313 | did not report outcome of interest | Salma, Roaa |
| Clinical profiling of stroke | 2017 | Bahrain Medical Bulletin | | 39 | 3 | 162-164 | Abdulsalam, A. M. M. and Alarab, M. M. E. and Afifi, K. K. and Al-Hussain, Y. F. and Al-Sulaiman, W. A. and Al-Sayyad, A. S. | https://www.scopus.com/inward/record.uri?eid=2-s2.0-85028354278&doi=10.12816%2f0047759&partnerID=40&md5=b30aaae48dd2ef8b05b425ed7250abcd | | | 10.12816/0047759 | full text not found | Salma, Roaa |
| Trends in stroke admissions before, during and post-peak of the COVID-19 pandemic: A one-year experience from the Qatar stroke database | 2022 | PLoS ONE |  | 17 | 3 |  | Akhtar, N. and Kamran, S. and Al-Jerdi, S. and Imam, Y. and Joseph, S. and Morgan, D. and Abokersh, M. and Uy, R. T. and Shuaib, A. | https://www.scopus.com/inward/record.uri?eid=2-s2.0-85126929609&doi=10.1371%2fjournal.pone.0255185&partnerID=40&md5=0ebc88a8d541b07dbd5b815ecd556035 | | | 10.1371/journal.pone.0255185 | did not report outcome of interest | SALMA, Salma, Roaa |
| Ethnic diversity and acute stroke features - A comparison from stroke registry qatar | 2016 | European Stroke Journal | 2396-9881 | 1 | 1 | 358 | Akhtar, N. and Kamran, S. and Bourke, P. and Joseph, S. and Santos, M. and Salam, A. and Deleu, D. and Shuaib, A. | https://www.embase.com/search/results?subaction=viewrecord&id=L616987894&from=export http://dx.doi.org/10.1177/2396987316642909 | English | N. Akhtar, Hamad General Hospital, NEUROSCIENCE, Doha, Qatar | 10.1177/2396987316642909 | conference abstract | SALMA, Salma, Roaa |
| Ischaemic posterior circulation stroke in State of Qatar | 2009 | European Journal of Neurology | | 16 | 9 | 1004-1009 | Akhtar, N. and Kamran, S. I. and Deleu, D. and D'Souza, A. and Miyares, F. and Elsotouhy, A. and Al-Hail, H. and Mesraoua, B. and Own, A. and Salem, K. and Kamha, A. and Osman, Y. | https://www.scopus.com/inward/record.uri?eid=2-s2.0-68849097067&doi=10.1111%2fj.1468-1331.2009.02709.x&partnerID=40&md5=07d8003f1c83e5feaf279cf174c37246 | | | 10.1111/j.1468-1331.2009.02709.x | did not report outcome of interest | Salma, Roaa |
| Ethnic variation in acute cerebrovascular disease: Analysis from the Qatar stroke registry | 2016 | European Stroke Journal | | 1 | 3 | 231-241 | Akhtar, N. and Salam, A. and Kamran, S. and Bourke, P. and Joseph, S. and Santos, M. and Khan, R. and Irfan, F. and Deleu, D. and Malik, R. A. and Shuaib, A. | https://www.scopus.com/inward/record.uri?eid=2-s2.0-85020509513&doi=10.1177%2f2396987316663776&partnerID=40&md5=f993cb704808815b429e2308b8168b3e | | | 10.1177/2396987316663776 | did not report outcome of interest | Salma, Roaa |
| STROKE IN AFRICA: A SYSTEMATIC REVIEW OF THE INCIDENCE, PREVALENCE AND FATALITY | 2022 | International Journal of Stroke | 1747-4949 | 17 | 3 | 116 | Akinyemi, R. and Okekunle, A. and Jones, S. and Watkins, C. and Hackett, M. | https://www.embase.com/search/results?subaction=viewrecord&id=L639619958&from=export http://dx.doi.org/10.1177/17474930221125973 | English | R. Akinyemi, University College Hospital, Department of Medicine, Ibadan, Nigeria | 10.1177/17474930221125973 | conference abstract | Salma, Roaa |
| Stroke in young adults: A 4-year retrospective hospital-based study, first report from United Arab Emirates | 2015 | Neurology | 0028-3878 | 84 |  |  | Al Ameri, M. and Al Nuaimi, A. and Alsaadi, T. | https://www.embase.com/search/results?subaction=viewrecord&id=L71920152&from=export | English | M. Al Ameri |  | conference abstract | Salma, Roaa |
| Stroke in Bahrain: Rising incidence, multiple risk factors, and suboptimal care | 2015 | International Journal of Stroke | | 10 | 4 | 615-618 | Al Banna, M. and Baldawi, H. and Kadhim, A. and Humaidan, H. and Whitford, D. L. | https://www.scopus.com/inward/record.uri?eid=2-s2.0-84929508393&doi=10.1111%2fijs.12513&partnerID=40&md5=94459b24a37da63954c74cf7c5e125e2 | | | 10.1111/ijs.12513 | can't be accessed | Salma, Roaa |
| Epidemiology of stroke the bahrain national stroke care audit: From acute event to hospital discharge | 2014 | International Journal of Stroke | 1747-4930 | 9 |  | 149 | Al Banna, M. and Baldawi, M. and Kadhim, A. and Whitford, D. | https://www.embase.com/search/results?subaction=viewrecord&id=L71646190&from=export http://dx.doi.org/10.1111/ijs.12375 | English | M. Al Banna, Department of Postgraduate Studies and Research, Royal College of Surgeons in Ireland - Bahrain, Medical University of Bahrain, Busaiteen, Bahrain | 10.1111/ijs.12375 | conference abstract | Salma, Roaa |
| Stroke subtypes and risk factors in Saudi Arabia | 2016 | Neurology | 0028-3878 | 86 | 16 |  | Al Harbi, A. and Shoamanesh, A. | https://www.embase.com/search/results?subaction=viewrecord&id=L72252235&from=export | English | A. Al Harbi |  | conference abstract | Salma, Roaa |
| Clinical Profile of Stroke Patients Presenting to the Emergency Department of a Major Stroke Centre in Oman | 2022 | Sultan Qaboos University Medical Journal | | 22 | 1 | 91-97 | Al Harthi, H. A. and Al Kashmiri, A. and Zakaryia, L. M. and Al-Lawati, J. A. and Najem, O. M. and Al-Lawati, I. and Memon, G. R. and Elfaham, A. A. | https://www.scopus.com/inward/record.uri?eid=2-s2.0-85125593847&doi=10.18295%2fsqumj.4.2021.062&partnerID=40&md5=7ff22f797b1c19bdedc85853d823bb42 | | | 10.18295/squmj.4.2021.062 | full text not found | Salma, Roaa |
| Large vessel occlusion stroke: Prevalence, predictors, and outcome. single tertiary care center study | 2020 | International Journal of Stroke | 1747-4949 | 15 | 1 | 397 | Al Khathaami, A. and Alskaini, M. and Alsaif, S. and Al Bdah, B. and Alhasson, M. and Aldriweesh, M. and Alluhidan, W. and Almutairi, F. and Alotaibi, J. and Alotaibi, N. and Alghamdi, S. | https://www.embase.com/search/results?subaction=viewrecord&id=L634009976&from=export http://dx.doi.org/10.1177/1747493020963387 | English | A. Al Khathaami, College of Medicine, King Saud Bin Abdul Aziz University for Health Sciences, Riyadh, Saudi Arabia | 10.1177/1747493020963387 | conference abstract | SALMA, Salma, Roaa |
| Embolic Stroke of Undetermined Source in Saudi Arabia: Prevalence, Patient Characteristics, and Outcomes | 2019 | Journal of Stroke and Cerebrovascular Diseases | | 28 | 12 |  | Al Khathaami, A. M. and Al Bdah, B. and Alnosair, A. and Alrebdi, R. and Alwayili, S. and Alhamzah, S. and Al Turki, A. and Alotaibi, N. | https://www.scopus.com/inward/record.uri?eid=2-s2.0-85073079548&doi=10.1016%2fj.jstrokecerebrovasdis.2019.104390&partnerID=40&md5=d4387ee6b81a7a342c4c80fdf17ebb1a | | | 10.1016/j.jstrokecerebrovasdis.2019.104390 | did not report outcome of interest | SALMA, Salma, Roaa |
| Stroke following acute coronary syndrome: Prevalence, predictors and outcome in six Middle-Eastern countries: Findings from the 2nd Gulf registry of acute coronary events (Gulf RACE-2) | 2012 | Circulation | 0009-7322 | 125 | 19 | e783 | Al Suwaidi, J. and Al Habib, K. and Asaad, N. and Singh, R. and Hersi, A. and Al Falaeh, H. and Al Saif, S. and Al-Motarreb, A. and Almahmeed, W. and Sulaiman, K. | https://www.embase.com/search/results?subaction=viewrecord&id=L71051547&from=export http://dx.doi.org/10.1161/CIR.0b013e31824fcdb3 | English | J. Al Suwaidi, Cardiology and cardiovascular surgery, Hamad Medical Corporation, Doha, Qatar | 10.1161/CIR.0b013e31824fcdb3 | conference abstract | SALMA, Salma, Roaa |
| Intracranial atherosclerotic disease among Saudis, prevalence, characteristics and predictors of outcome | 2019 | Journal of the Neurological Sciences | 1878-5883 0022-510X | 405 |  | 04-Mar | Alabbas, M. and Al Khathaami, A. and El Metwally, A. and Badri, M. and Alskaini, M. | https://www.embase.com/search/results?subaction=viewrecord&id=L2003651360&from=export http://dx.doi.org/10.1016/j.jns.2019.10.218 | English |  | 10.1016/j.jns.2019.10.218 | conference abstract | SALMA, Salma, Roaa |
| A snapshot of Ischemic stroke risk factors, sub-types, and its epidemiology: Cohort study | 2020 | Annals of Medicine and Surgery | | 59 |  | 101-105 | Alawneh, K. Z. and Al Qawasmeh, M. and Raffee, L. A. and Abuzayed, B. and Bani Hani, D. A. and Abdalla, K. M. and Al-Mnayyis, A. M. and Fataftah, J. | https://www.scopus.com/inward/record.uri?eid=2-s2.0-85091234532&doi=10.1016%2fj.amsu.2020.09.016&partnerID=40&md5=25ff6db36f1ff9298f3087a559a5e5b0 | | | 10.1016/j.amsu.2020.09.016 | did not report outcome of interest | Salma, Roaa |
| Early stroke following acute myocardial infarction: Incidence, predictors and outcome in six middle-eastern countries | 2011 | Cerebrovascular Diseases | | 32 | 5 | 471-482 | Albaker, O. and Zubaid, M. and Alsheikh-Ali, A. A. and Rashed, W. and Alanbaei, M. and Almahmeed, W. and Al-Shereiqi, S. Z. and Sulaiman, K. and Al Qahtani, A. and Al Suwaidi, J. | https://www.scopus.com/inward/record.uri?eid=2-s2.0-80155182247&doi=10.1159%2f000330344&partnerID=40&md5=350ff68c8b23581da2fad815f0756b4c | | | 10.1159/000330344 | did not report outcome of interest | Salma, Roaa |
| Cardioembolic stroke: Prevalence, patients characteristics and outcome in Saudis | 2019 | Journal of the Neurological Sciences | 1878-5883 0022-510X | 405 |  | 77 | Alboqami, Q. and Alskaini, M. and Badri, M. and El-Metwally, A. and Al Khathaami, A. | https://www.embase.com/search/results?subaction=viewrecord&id=L2003654763&from=export http://dx.doi.org/10.1016/j.jns.2019.10.573 | English |  | 10.1016/j.jns.2019.10.573 | conference abstract | Salma, Roaa |
| Prevalence of stroke and myocardial infarction among patients with deteriorated GFR | 2022 | European Review for Medical and Pharmacological Sciences | | 26 | 17 | 6259-6264 | Alharbi, S. H. | https://www.scopus.com/inward/record.uri?eid=2-s2.0-85138315887&doi=10.26355%2feurrev_202209_29649&partnerID=40&md5=d5111899e0161ec7b1fa721ed693bdb8 | | | 10.26355/eurrev_202209_29649 | did not report outcome of interest | Salma, Roaa, Duaa |
| Epidemiology and clinical characteristics of stroke in young Egyptian adults | 2023 | European Stroke Journal | 2396-9881 | 8 | 2 | 521 | Ali, A. and Ahmed, M. A. R. and Shehab, M. M. and Abdelhaleem, M. A. and Lioutas, V. A. | https://www.embase.com/search/results?subaction=viewrecord&id=L641735197&from=export http://dx.doi.org/10.1177/23969873231169660 | English | A. Ali, Assiut University, Neurology, Assiut, Egypt | 10.1177/23969873231169660 | conference abstract | Salma, Roaa |
| Epidemiology of cerebral ischemic stroke in southern region in Saudi Arabia and expected role of neurosurgery | 2020 | Neurology | 1526-632X | 94 | 15 |  | Alkhayri, M. | https://www.embase.com/search/results?subaction=viewrecord&id=L633067658&from=export | English | M. Alkhayri, College of Medicine, King Khalid University | | conference abstract | Salma, Roaa |
| The prevalence of macrovascular complications among diabetic patients in the United Arab Emirates | 2007 | Cardiovascular diabetology | | 6 |  | 24 | Al-Maskari, F. and El-Sadig, M. and Norman, J. N. | https://www.scopus.com/inward/record.uri?eid=2-s2.0-38449089314&doi=10.1186%2f1475-2840-6-24&partnerID=40&md5=461feacc5819d1e4d5025e264ea677f6 | | | 10.1186/1475-2840-6-24 | did not report outcome of interest | Salma, Roaa, Duaa |
| Incidence and impact of stroke during Hajj. Results of 2015 Hajj stroke registry | 2017 | Neurosciences |  | 22 | 3 | 181-185 | Almekhlafi, M. A. and Alhazmi, M. A. and Alsulami, S. S. and Almorsy, S. A. | https://www.scopus.com/inward/record.uri?eid=2-s2.0-85021758094&doi=10.17712%2fnsj.2017.3.20160246&partnerID=40&md5=ed735b15987d73aeabb9a8a965a068dd | | | 10.17712/nsj.2017.3.20160246 | did not report outcome of interest | Salma, Roaa, Duaa |
| The prevalence and risk factors of stroke among Sudanese individuals with diabetes: Cross-sectional survey | 2020 | Brain Circ | 2394-8108 (Print) 2394-8108 | 6 | 1 | 26-30 | Almobarak, A. O. and Badi, S. and Elmadhoun, W. M. and Tahir, H. and Ahmed, M. H. | | eng | Department of Pathology, Faculty of Medicine, University of Medical Sciences and Technology, Omdurman, Khartoum, Sudan. Delta College of Science and Technology, Faculty of Pharmacy, Omdurman Islamic University, Omdurman, Khartoum, Sudan. Department of Clinical Pharmacy, Faculty of Pharmacy, Omdurman Islamic University, Omdurman, Khartoum, Sudan. Department of Pathology, Faculty of Medicine, Nile Valley University, Atbara, Sudan. Public and Tropical Health Program, Graduate College, University of Medical Sciences and Technology, Khartoum, Sudan. Department of Medicine and HIV Metabolic Clinic, Milton Keynes University Hospital NHS Foundation Trust, Eaglestone, Milton Keynes, Buckinghamshire, UK. | 10.4103/bc.bc_15_19 | did not report outcome of interest | Salma, Roaa |
| Incidence of stroke among Saudi population: a systematic review and meta-analysis | 2020 | Neurological Sciences |  | 41 | 11 | 3099-3104 | Alqahtani, B. A. and Alenazi, A. M. and Hoover, J. C. and Alshehri, M. M. and Alghamdi, M. S. and Osailan, A. M. and Khunti, K. | https://www.scopus.com/inward/record.uri?eid=2-s2.0-85086771298&doi=10.1007%2fs10072-020-04520-4&partnerID=40&md5=0927aeaecdffc4f004bac3afec32b705 | | | 10.1007/s10072-020-04520-4 | did not report outcome of interest | Salma, Roaa |
| Incidence, predictors, and mortality of in-hospital stroke after acute coronary syndrome in the Middle East | 2020 | Annals of Clinical Cardiology | 2666-6987 2666-6979 | 2 | 1 | 13-18 | Alsaeed, W. and Al-Zakwani, I. and Panduranga, P. and Zubaid, M. and Rashed, W. and Brady, P. A. | https://www.embase.com/search/results?subaction=viewrecord&id=L632444465&from=export http://dx.doi.org/10.4103/ACCJ.ACCJ_4_20 | English | W. Alsaeed, Department of Cardiology, Sabah Al Ahmad Cardiac Center, Al Amiri Hospital, Kuwait City, Kuwait | 10.4103/ACCJ.ACCJ_4_20 | full text not found | Salma, Roaa |
| An Epidemiological Model for First Stroke in Saudi Arabia | 2020 | Journal of Stroke and Cerebrovascular Diseases | | 29 | 1 |  | Al-Senani, F. and Al-Johani, M. and Salawati, M. and Alhazzani, A. and Morgenstern, L. B. and Seguel Ravest, V. and Cuche, M. and Eggington, S. | https://www.scopus.com/inward/record.uri?eid=2-s2.0-85075353547&doi=10.1016%2fj.jstrokecerebrovasdis.2019.104465&partnerID=40&md5=acb41a9cc74d1ce13f891f5b9540b7ec | | | 10.1016/j.jstrokecerebrovasdis.2019.104465 | did not report outcome of interest | Salma, Roaa, Duaa |
| First stroke incidence in Saudi Arabia over the next decade: Results for The Riyadh Region | 2019 | European Stroke Journal | 2396-9881 | 4 |  | 315-316 | Al-Senani, F. and Salawati, M. and AlJohani, M. and Cuche, M. and Seguel Ravest, V. and Eggington, S. | https://www.embase.com/search/results?subaction=viewrecord&id=L628561690&from=export http://dx.doi.org/10.1177/2396987319845581 | English | F. Al-Senani, National Neurosciences Institute, King Fahad Medical City, Department of Neurology, Riyadh, Saudi Arabia | 10.1177/2396987319845581 | conference abstract | Salma, Roaa, Duaa |
| Replay: Prevalence of silent stroke in Kurdistan, Iraq | 2011 | Neurosciences |  | 16 | 1 | 86 | Al-Shimmery, E. K. | https://www.scopus.com/inward/record.uri?eid=2-s2.0-79951936410&partnerID=40&md5=e3f274ee3fea43ae2c3d8633d67b3a27 | | | | Letter | Salma, Roaa, Duaa |
| Stroke in women: Results from the Mashhad Stroke Incidence Study (MSIS), a population based study in the Middle- East | 2017 | Neurology | 1526-632X | 88 | 16 |  | Amiri, A. and Azarpazhooh, M. R. and Saber, H. and Shoamanesh, A. and Behrouz, R. | https://www.embase.com/search/results?subaction=viewrecord&id=L616550856&from=export | English | A. Amiri, Neurology, Mashhad University of Medical Sciences, Mashhad, Iran | | conference abstract | Salma, Roaa |
| Epidemiology of stroke in sohag; hospital based study | 2020 | QJM | 1460-2393 | 113 |  | i148 | Aref, H. and Ahmed, S. and Shalash, A. and Abdelrahem, M. I. A. | https://www.embase.com/search/results?subaction=viewrecord&id=L636768840&from=export http://dx.doi.org/10.1093/qjmed/hcaa054.014 | English | M.I.A. Abdelrahem, Department of Neurology, Faculty of Medicine, Ain Shams University, Cairo, Egypt | 10.1093/qjmed/hcaa054.014 | conference abstract | Salma, Roaa, Duaa |
| Excessive incidence of stroke in Iran: Evidence from the Mashhad stroke incidence study (MSIS), a population-based study of stroke | 2010 | International Journal of Stroke | 1747-4930 | 5 |  | 207 | Azarpazhooh, M. R. and Etemadi, M. M. and Donnan, G. A. and Mokhber, N. and Majdi, M. R. and Ghayour-Mobarhan, M. and Ghandehary, K. and Farzadfard, M. T. and Kiani, R. and Panahandeh, M. and Thrift, A. G. | https://www.embase.com/search/results?subaction=viewrecord&id=L70335393&from=export http://dx.doi.org/10.1111/j.1747-4949.2010.00480.x | English | M.R. Azarpazhooh, Department of Neurology, Ghaem Hospital, Mashhad University of Medical Sciences, Mashhad, Iran | 10.1111/j.1747-4949.2010.00480.x | conference abstract | Salma, Roaa |
| Age, sex and stroke type differences in stroke patients at Mukalla, Hadhramout, Republic of Yemen: Analysis of 1072 cases | 2013 | Journal of the Neurological Sciences | 0022-510X | 333 |  | e263 | Bamekhlah, R. M. and Al Ghazali, H. S. and Musaian, N. S. | https://www.embase.com/search/results?subaction=viewrecord&id=L71188579&from=export http://dx.doi.org/10.1016/j.jns.2013.07.1008 | English | R.M. Bamekhlah, Internal Medicine, College of Medicine, Hadhramout University, Mukalla City, Yemen | 10.1016/j.jns.2013.07.1008 | conference abstract | Salma, Roaa |
| Burden of stroke in the Kingdom of Saudi Arabia: A soaring epidemic | 2021 | Saudi Pharmaceutical Journal | | 29 | 3 | 264-268 | Basri, R. and Issrani, R. and Hua Gan, S. and Prabhu, N. and Khursheed Alam, M. | https://www.scopus.com/inward/record.uri?eid=2-s2.0-85101414969&doi=10.1016%2fj.jsps.2021.02.002&partnerID=40&md5=643952a3de2beb71e5185ac59d60d8bb | | | 10.1016/j.jsps.2021.02.002 | did not report outcome of interest | Salma, Roaa |
| Stroke in Arab countries: A systematic literature review | 2009 | Journal of the Neurological Sciences | | 284 | 1 | 18-23 | Benamer, H. T. and Grosset, D. | https://www.scopus.com/inward/record.uri?eid=2-s2.0-67849119440&doi=10.1016%2fj.jns.2009.04.029&partnerID=40&md5=4048f2520747428042a1ddb511c246f8 | | | 10.1016/j.jns.2009.04.029 | wrong study duration | Salma, Roaa, Duaa |
| Stroke in the Blida region (Algeria) in 2018: Incidence and mortality according to a population-based register | 2020 | Tunisie Medicale |  | 98 | 1 | 16-Aug | Bezzaoucha, A. and Bouamra, A. and Zeddam, F. and Ammimer, A. and Kadi, F. and Abdi, N. and Akrour, Z. and Kesraoui, S. and Arezki, M. and Abdelaziz, A. B. | https://www.scopus.com/inward/record.uri?eid=2-s2.0-85083719024&partnerID=40&md5=dd0477d151db145097bc56e7ec85d6d1 | | | | can't be accessed | SALMA, Salma, Duaa |
| A comprehensive epidemiology of stroke in a multi ethnic society: An analysis of a nationwide stroke data from 2014-2020 | 2022 | Stroke | 1524-4628 | 53 |  |  | Bhutta, Z. A. and Akhtar, N. and Pathan, S. A. and Puolakka, T. and Harris, T. and Ganesan, G. S. S. and Kamran, S. and Thomas, S. H. and Cameron, P. and Castren, M. K. | https://www.embase.com/search/results?subaction=viewrecord&id=L637365685&from=export http://dx.doi.org/10.1161/str.53.suppl_1.TP195 | English | Z.A. Bhutta | 10.1161/str.53.suppl_1.TP195 | conference abstract | SALMA, Roaa, Duaa |
| Incdence of ischemic stroke in constantine city | 2017 | Cerebrovascular Diseases | 1421-9786 | 43 |  | 37 | Boubekeur Saddik, F. and Abdarrahim, M. and Fatima, S. | https://www.embase.com/search/results?subaction=viewrecord&id=L619777524&from=export | English | F. Boubekeur Saddik, Faculte de Medecine Constante. Algerie, Constantine, Algeria | | conference abstract | SALMA, Roaa, Duaa |
| Epidemiology study of stroke about 1256 cases | 2013 | Journal of the Neurological Sciences | 0022-510X | 333 |  | e248 | Bourazza, A. and Hsaini, Y. | https://www.embase.com/search/results?subaction=viewrecord&id=L71188531&from=export http://dx.doi.org/10.1016/j.jns.2013.07.960 | English | A. Bourazza, Neurology of Military Hospital Mohammed V Souissi, Rabat, Morocco | 10.1016/j.jns.2013.07.960 | conference abstract | Salma, Roaa |
| Risk factors for ischemic stroke, about 442 cases | 2017 | Journal of the Neurological Sciences | 1878-5883 | 381 |  | 403-404 | Chraa, M. and Chaqda, M. and Najib | https://www.embase.com/search/results?subaction=viewrecord&id=L620183931&from=export http://dx.doi.org/10.1016/j.jns.2017.08.3353 | English | M. Chraa, Mohammed VI University Hospital, Neurology/Physiology, Marrakech, Morocco | 10.1016/j.jns.2017.08.3353 | conference abstract | Salma, Roaa |
| Epidemiology of stroke in Shiraz, Iran | 2015 | Iranian Journal of Neurology | 2252-0058 2008-384X | 14 | 3 | 158-163 | Daneshfard, B. and Izadi, S. and Shariat, A. and Toudaji, M. A. and Beyzavi, Z. and Niknam, L. | https://www.embase.com/search/results?subaction=viewrecord&id=L605380107&from=export | English | A. Shariat, Shiraz Neuroscience Research Center, Clinical Neurology Research Center, Department of Neurology, Shiraz University of Medical Sciences, Shiraz, Iran | | did not report outcome of interest | SALMA, Duaa |
| Stroke epidemiology and one-month fatality in an Iranian city | 2009 | Cerebrovascular Diseases | 1015-9770 | 27 |  | 97 | Delbari, A. and Lokk, J. and Rahgozar, M. and Tabatabaei, S. S. and Roghani, R. S. | https://www.embase.com/search/results?subaction=viewrecord&id=L70017994&from=export http://dx.doi.org/10.1159/000221776 | English | A. Delbari, Karolinska Institute, Stockholm, Sweden | 10.1159/000221776 | conference abstract | Salma, Roaa |
| Stroke epidemiology and one-month fatality among an urban population in Iran | 2011 | International Journal of Stroke | | 6 | 3 | 195-200 | Delbari, A. and Salman Roghani, R. and Tabatabaei, S. S. and Rahgozar, M. and Lokk, J. | https://www.scopus.com/inward/record.uri?eid=2-s2.0-79955763227&doi=10.1111%2fj.1747-4949.2010.00562.x&partnerID=40&md5=6205a131c6a6c4585e8010609eabc2fe | | | 10.1111/j.1747-4949.2010.00562.x | did not report outcome of interest | Salma, Roaa |
| Stroke in-hospital survival and its predictors: The first results from Tabriz stroke registry of Iran | 2018 | International Journal of General Medicine | | 11 |  | 233-240 | Deljavan, R. and Farhoudi, M. and Sadeghi-Bazargani, H. | https://www.scopus.com/inward/record.uri?eid=2-s2.0-85049200285&doi=10.2147%2fIJGM.S158296&partnerID=40&md5=6f840058870e79a95fdc7fe887bf21e8 | | | 10.2147/IJGM.S158296 | did not report outcome of interest | SALMA, Salma, Roaa |
| Global, Regional, and National Burden of Ischemic Stroke, 1990'2019 | 2022 | Neurology |  | 98 | 3 | E279-E290 | Ding, Q. and Liu, S. and Yao, Y. and Liu, H. and Cai, T. and Han, L. | https://www.scopus.com/inward/record.uri?eid=2-s2.0-85123651082&doi=10.1212%2fWNL.0000000000013115&partnerID=40&md5=0d1775375dca4242fae01bc8c2ff931f | | | 10.1212/WNL.0000000000013115 | full text not found | Salma, Roaa |
| Ethnic differences in stroke outcomes in the state of Qatar | 2015 | International Journal of Stroke | 1747-4930 | 10 |  | 203 | D'Souza, A. and Shuaib, A. and Akhtar, N. and Bourke, P. and Joseph, S. and Santos, M. and Imam, Y. Z. and Kamran, S. and Deleu, D. | https://www.embase.com/search/results?subaction=viewrecord&id=L72034246&from=export http://dx.doi.org/10.1111/ijs.12479 | English | A. D'souza, Medicine/Neurology, Hamad General Hospital, Doha, Qatar | 10.1111/ijs.12479 | conference abstract | SALMA, Salma, Roaa |
| Epidemiology of intracerebral hemorrhage in Qatar | 2018 | Stroke | 1524-4628 | 49 |  |  | Dsouza, A. and Shuaib, A. and Kamran, S. and Akhtar, N. and El Sotouhy, A. and Abbas, S. and Ali, M. and Bourke, P. and Joseph, S. and Saqqur, M. | https://www.embase.com/search/results?subaction=viewrecord&id=L621005342&from=export | English | A. Dsouza, Hamad Med Corp, Doha, Qatar | | conference abstract | Salma, Roaa |
| The epidemiology of stroke in the Middle East | 2016 | European Stroke Journal | | 1 | 3 | 180-198 | El-Hajj, M. and Salameh, P. and Rachidi, S. and Hosseini, H. | https://www.scopus.com/inward/record.uri?eid=2-s2.0-85020548391&doi=10.1177%2f2396987316654338&partnerID=40&md5=b6b38d53b50d6320bfe71a89bacd774f | | | 10.1177/2396987316654338 | did not report outcome of interest | SALMA, Salma, Roaa |
| A systematic review of stroke in the Middle East | 2016 | Cerebrovascular Diseases | 1421-9786 | 41 |  | 107 | El-Hajj, M. and Salameh, P. and Rachidi, S. and Hosseini, H. | https://www.embase.com/search/results?subaction=viewrecord&id=L72340765&from=export | English | M. El-Hajj, Universite Paris-Est, Creteil, France | | conference abstract | SALMA, Salma, Roaa |
| Stroke in young: Aetiologies, pattern of presentation, demographic and imaging characteristics | 2021 | Journal of the Neurological Sciences | 1878-5883 0022-510X | 429 |  |  | Elmahal, M. | https://www.embase.com/search/results?subaction=viewrecord&id=L2014980595&from=export http://dx.doi.org/10.1016/j.jns.2021.119703 | English |  | 10.1016/j.jns.2021.119703 | conference abstract | SALMA, Duaa |
| Stroke in Egypt | 2010 | Revue Neurologique | 0035-3787 | 166 | 3 | 355 | El-Tamawy, M. | https://www.embase.com/search/results?subaction=viewrecord&id=L70133234&from=export http://dx.doi.org/10.1016/j.neurol.2009.07.016 | English | M. El-Tamawy, Head of Stroke Unit, Cairo University, Egypt | 10.1016/j.neurol.2009.07.016 | conference abstract | Salma, Roaa |
| Neurological events in children with infective endocarditis: Results from a prospective cohort | 2020 | Archives of Cardiovascular Diseases Supplements | 1878-6502 1878-6480 | 12 | 1 | 96-97 | Farah, A. and Ben Abdeljelil, O. and Jomaa, W. and Chamtouri, I. and Ben Hamda, K. and Maatouk, F. | https://www.embase.com/search/results?subaction=viewrecord&id=L2003962914&from=export http://dx.doi.org/10.1016/j.acvdsp.2019.09.211 | English | A. Farah, Fattouma-Bourguiba Hospital, Monastir, Tunisia | 10.1016/j.acvdsp.2019.09.211 | conference abstract | Salma, Roaa, Duaa |
| Prevalence of stroke symptoms among stroke-free residents: National data from Lebanon | 2015 | European Journal of Neurology | 1351-5101 | 22 |  | 208 | Farah, R. and Zeidan, R. K. and Chahine, M. and Chahine, R. and Salameh, P. and Hosseini, H. | https://www.embase.com/search/results?subaction=viewrecord&id=L71933445&from=export http://dx.doi.org/10.1111/ene.12807 | English | R. Farah, Henri Mondor Hospital, Neurology, Paris, France | 10.1111/ene.12807 | did not report outcome of interest | SALMA, Duaa |
| Study on epidemiology and risk factors of ischemic and hemorrhagic stroke in northwest Iran, 2009 | 2011 | Cerebrovascular Diseases | 1015-9770 | 31 |  | 211 | Farhoudi, M. and Pashapour, A. and Ladan, A. and Mostafaie, S. and Sharifipour, E. and Sadeghi-Hokmabadi, E. | https://www.embase.com/search/results?subaction=viewrecord&id=L70432826&from=export http://dx.doi.org/10.1159/000329448 | English | M. Farhoudi, Neuroscience Research Center (NSRC), Tabriz University of Medical Sciences, Tabriz, Iran | 10.1159/000329448 | conference abstract | Salma, Roaa |
| 25: Stroke in the region - Stroke epidemiology, diagnosis and treatment options, regional differences and future suggestions for collaboration stroke in Iran | 2014 | International Journal of Stroke | 1747-4930 | 9 |  | 4 | Farhoudi, M. and Sadeghi-Hokmabad, E. and Sharifipour, E. and Mehrvar, K. and Taheraghdam, A. and Hasmilar, M. and Savadi Oskoui, D. and Yazdchi Marandi, M. and Ayromlou, H. and Pashapour, A. and Gheini, M. R. and Mehrpour, M. and Borhanhaghighi, A. and Ghandhari, K. and Khorvash, F. | https://www.embase.com/search/results?subaction=viewrecord&id=L71645793&from=export http://dx.doi.org/10.1111/ijs.12375 | English | M. Farhoudi, Neurology, Neurosciences Research Center, Tabriz University of Medical Sciences, Tabriz, Iran | 10.1111/ijs.12375 | conference abstract | Salma, Roaa |
| Statistical comparison of stroke patients visiting an emergency department during summer versus winter: A cross-sectional study | 2020 | Archives of Neuroscience | 2322-5769 2322-3944 | 7 | 2 |  | Forouzanfar, M. M. and Sepehrirad, A. and Heydari, K. and Mirbaha, S. | https://www.embase.com/search/results?subaction=viewrecord&id=L2004362164&from=export http://dx.doi.org/10.5812/ans.101946 | English | S. Mirbaha, Department of Emergency Medicine, Shohada-e-Tajrish Hospital, Shahid Beheshti University of Medical Sciences, Tehran, Iran | 10.5812/ans.101946 | can't be accessed | SALMA, Duaa |
| Epidemiology of stroke in Iran | 2016 | Galen Medical Journal |  | 5 |  | 09-Mar | Ghandehari, K. | https://www.scopus.com/inward/record.uri?eid=2-s2.0-85071699982&partnerID=40&md5=9f95c1805a4f0ad225d110946a6f71db | | | | Letter | SALMA, Duaa |
| Khorasan posterior circulation stroke registry: A hospital-based study | 2008 | Iranian Journal of Medical Sciences | | 33 | 2 | 67-73 | Ghandehari, K. and Etemadi, M. M. and Nikrad, M. and Shakeri, M. T. and Mansoori, M. | https://www.scopus.com/inward/record.uri?eid=2-s2.0-48649085732&partnerID=40&md5=f56077283d578093479e0c12d4d82ce1 | | | | did not report outcome of interest | SALMA, Roaa, Duaa |
| The Khorasan Stroke Registry: Results of a five-year hospital-based study | 2007 | Cerebrovascular Diseases | | 23 | 2 | 132-139 | Ghandehari, K. and Izadi, Z. | https://www.scopus.com/inward/record.uri?eid=2-s2.0-33845717433&doi=10.1159%2f000097050&partnerID=40&md5=2d9386a636d75f2f052384f6a972f6d5 | | | 10.1159/000097050 | did not report outcome of interest | SALMA, Duaa |
| Etiology of young adult onset brain infarction in Iran | 2006 | Archives of Iranian Medicine | | 9 | 3 | 240-243 | Ghandehari, K. and Izadi-Mood, Z. | https://www.scopus.com/inward/record.uri?eid=2-s2.0-33746467881&partnerID=40&md5=dbe6c20801354a1e252055a5d8d703c6 | | | | wrong study duration | SALMA, Duaa |
| Stroke Care Trends During COVID-19 Pandemic in Zanjan Province, Iran. From the CASCADE Initiative: Statistical Analysis Plan and Preliminary Results | 2020 | Journal of Stroke & Cerebrovascular Diseases | 1052-3057 | 29 | 12 | N.PAG-N.PAG | Ghoreishi, Abdoreza and Arsang-Jang, Shahram and Sabaa-Ayoun, Ziad and Yassi, Nawaf and Sylaja, P. N. and Akbari, Yama and Divani, Afshin A. and Biller, Jose and Phan, Thanh and Steinwender, Sandy and Silver, Brian and Zand, Ramin and Basri, Hamidon Bin and Iqbal, Omer M. and Ranta, Annemarei and Ruland, Sean and Macri, Elizabeth and Ma, Henry and Nguyen, Thanh N. and Abootalebi, Shahram | https://search.ebscohost.com/login.aspx?direct=true&db=cul&AN=147070877&site=ehost-live&scope=site&authtype=sso&custid=s3704754 | | Stroke Research Group, Department of Neurology and Stroke Unit, Vali-e-Asr hospital, School of Medicine, Zanjan University of Medical Sciences, Zanjan, Iran Department of Biostatistics and Epidemiology, School of Medicine, Zanjan University of Medical Sciences, Zanjan, Iran Stroke Prevention and Atherosclerosis Research Centre, Robarts Research Institute, Western University, London, Ontario, Canada Schulich School of Medicine and Dentistry, Western University, London, Ontario, Canada Department of Medicine and Neurology, Melbourne Brain Centre at The Royal Melbourne Hospital, University of Melbourne, Australia Population Health and Immunity Division, The Walter and Eliza Hall Institute of Medical Research, Parkville, Australia Comprehensive Stroke Care Program, Sree Chitra Tirunal Institute for Medical Sciences and Technology (SCTIMST), Trivandrum, Kerala, India Departments of Neurology, Neurological Surgery, and Anatomy & Neurobiology, Beckman Laser Institute & Medical Clinic, University of California, Irvine, CA, USA Department of Neurology, School of Medicine, University of New Mexico, Albuquerque, NM, USA Department of Neurology, Loyola University, Stritch School of Medicine, Chicago, IL, USA Department of Neurology, Monash Health and Department of Medicine, School of Clinical Sciences, Monash University, Australia Health Information Science, Western University, London, Ontario, Canada Regional Stroke Prevention, Southwestern Ontario Stroke Network, London Health Sciences, London, Ontario, Canada Department of Neurology, University of Massachusetts Medical School, Worcester, MA, USA Department of Neurology, Neuroscience Institute, Geisinger Medical Center, Danville, PA, USA Department of Medicine, Faculty of Medicine and Health Sciences, UPM, Serdang, Malaysia Department of Pathology and Ophthalmology, Center for Translational Research & Education, Loyola University Stritch School of Medicine, Maywood, IL, USA Department of Medicine and Neurology, University of Otago and Wellington Hospital, Wellington, New Zealand Boston Medical Center, Boston University School of Medicine, Boston, MA, USA Dr. Everett Chalmers Regional Hospital, Dalhousie University, New Brunswick, Canada | 10.1016/j.jstrokecerebrovasdis.2020.105321 | did not report outcome of interest | SALMA, Salma, Duaa |
| Shedding Light on the Causes and Characteristics of Stroke in Lebanon: A Systematic Review of Literature | 2022 | Journal of Geriatric Psychiatry and Neurology | | 35 | 5 | 655-662 | Gifford, A. and Biffi, A. and Gelaye, B. and Chemali, Z. | https://www.scopus.com/inward/record.uri?eid=2-s2.0-85115675817&doi=10.1177%2f08919887211044753&partnerID=40&md5=d7703a0ed587e7063388a232d16dec79 | | | 10.1177/08919887211044753 | can't be accessed | SALMA, Duaa |
| EPIDEMIOLOGY OF STROKE AT ORAN UNIVERSITY HOSPITAL (ALGERIA) | 2022 | European Stroke Journal | 2396-9881 | 7 | 1 | 223-224 | Goulmane, M. | https://www.embase.com/search/results?subaction=viewrecord&id=L638375066&from=export http://dx.doi.org/10.1177/23969873221087559 | English | M. Goulmane, Faculty of Medicine of Oran, Medecine, Oran, Algeria | 10.1177/23969873221087559 | conference abstract | SALMA, Duaa |
| Genetics of stroke syndromes | 2011 | Egyptian Journal of Neurology, Psychiatry and Neurosurgery | | 48 | 1 | 09-Mar | Hamdy, S. M. | https://www.scopus.com/inward/record.uri?eid=2-s2.0-79957667036&partnerID=40&md5=5f4aa6873b4477dd376a18f8f0c258e0 | | | | did not report outcome of interest | SALMA, Duaa |
| Investigating the Seasonal Pattern of Stroke Incidence and the Association Between Daily Stroke Occurrences and Meteorological Factors | 2014 | Journal of Guilan University of Medical Sciences | 2008-4048 | 23 | 90 | 50-58 | Hosininezhad, M. and Bakhshayesh, B. and Moaddabi, Y. and Hatamyan, H. R. | https://search.ebscohost.com/login.aspx?direct=true&db=cul&AN=102974246&site=ehost-live&scope=site&authtype=sso&custid=s3704754 | | Department of Neurology, Hospital Poorsina, School of Medicine, Guilan University of Medical sciences, Rasht, Iran | | did not report outcome of interest | SALMA, Duaa |
| Stroke in iran: A systematic review | 2009 | Journal of Neurology | 0340-5354 | 256 |  | S179 | Hosseini, A. and Benamer, H. | https://www.embase.com/search/results?subaction=viewrecord&id=L70019530&from=export http://dx.doi.org/10.1007/s00415-009-5161-z | English | A. Hosseini, Royal Wolverhampton Hospital NHS Trust, Wolverhampton, United Kingdom | 10.1007/s00415-009-5161-z | conference abstract | Salma, Duaa |
| Burden of non-communicable diseases in Iraq after the 2003 war | 2019 | Saudi Medical Journal |  | 40 | 1 | 72-78 | Hussain, A. M. and Lafta, R. K. | https://www.scopus.com/inward/record.uri?eid=2-s2.0-85059926220&doi=10.15537%2fsmj.2019.1.23463&partnerID=40&md5=4102fc31cf27da5cca1b345048a63c8a | | | 10.15537/smj.2019.1.23463 | did not report outcome of interest | SALMA, Duaa |
| Burden of Stroke in Qatar | 2015 | Journal of Stroke and Cerebrovascular Diseases | | 24 | 12 | 2875-2879 | Ibrahim, F. and Deleu, D. and Akhtar, N. and Al-Yazeedi, W. and Mesraoua, B. and Kamran, S. and Shuaib, A. | https://www.scopus.com/inward/record.uri?eid=2-s2.0-84949638022&doi=10.1016%2fj.jstrokecerebrovasdis.2015.08.024&partnerID=40&md5=a59d46704441edcda5f62ef0974eeb38 | | | 10.1016/j.jstrokecerebrovasdis.2015.08.024 | did not report outcome of interest | Salma, Roaa |
| Stroke in the adult Qatari population (Q-stroke) a hospital based retrospective observational cohort study | 2020 | International Journal of Stroke | 1747-4949 | 15 | 1 | 386 | Imam, Y. and Kamran, S. and Al Jerdi, S. and Chandra, P. and Alkhawad, N. and Saqqur, M. and Akhtar, N. and Ibrahim, F. and Elkhider, H. and Deleu, D. and Perkins, J. and Shuaib, A. and Abdelmoneim, M. | https://www.embase.com/search/results?subaction=viewrecord&id=L634005627&from=export http://dx.doi.org/10.1177/1747493020963387 | English | Y. Imam, Hamad Medical Corporation, Neurology, Qatar | 10.1177/1747493020963387 | did not report outcome of interest | SALMA, Duaa |
| Current status of stroke in Qatar: Including data from the BRAINS study | 2019 | JRSM Cardiovascular Disease | 2048-0040 | 8 |  |  | Jallow, E. and Al Hail, H. and Han, T. S. and Sharma, S. and Deleu, D. and Ali, M. and Al Hussein, H. and Abuzaid, H. O. and Sharif, K. and Khan, F. Y. and Sharma, P. | https://www.embase.com/search/results?subaction=viewrecord&id=L628962931&from=export http://dx.doi.org/10.1177/2048004019869160 | English | P. Sharma, Institute of Cardiovascular Research, Royal Holloway University of London, London, United Kingdom | 10.1177/2048004019869160 | did not report outcome of interest | SALMA, Duaa |
| The urgent need of primary prevention of stroke in children with sickle cell disease in Saudi Arabia using transcranial Doppler | 2013 | Haematologica | 0390-6078 | 98 |  | 675 | Jaouni, S. and Hammad, R. and Qarni, N. and Raffa, I. | https://www.embase.com/search/results?subaction=viewrecord&id=L71697561&from=export | English | S. Jaouni, Hematology Department, Consultant of Hematology, King Abdul Aziz University, Jeddah, Saudi Arabia | | conference abstract | SALMA, Duaa |
| Young ischemic stroke in Tunisia: a multicentric study | 2017 | International Journal of Neuroscience | | 127 | 4 | 314-319 | Kefi, A. and Larbi, T. and Abdallah, M. and Ouni, A. E. and Bougacha, N. and Bouslama, K. and Hamzaoui, S. and M'Rad, S. | https://www.scopus.com/inward/record.uri?eid=2-s2.0-84994877225&doi=10.1080%2f00207454.2016.1214131&partnerID=40&md5=6d2fe98127a1634581f8b55a85d1555a | | | 10.1080/00207454.2016.1214131 | wrong study duration | SALMA, Salma |
| Epidemiological study and outcome of acute stroke in the province of blida (Algeria) | 2011 | European Journal of Neurology | 1351-5101 | 18 |  | 374 | Kesraoui, S. and Arezki, M. and Boutarene, N. | https://www.embase.com/search/results?subaction=viewrecord&id=L70603035&from=export http://dx.doi.org/10.1111/j.1468-1331.2011.03552.x | English | S. Kesraoui, University of Blida, Blida, Algeria | 10.1111/j.1468-1331.2011.03552.x | conference abstract | SALMA, Duaa |
| Prevalence and Co-prevalence of Comorbidities Among Patients with Type 2 Diabetes Mellitus in the MENA Region: A Systematic Review | 2023 | Current diabetes reviews | 1875-6417 |  |  |  | Khalil, S. A. and Azar, S. and Hafidh, K. and Ayad, G. and Safwat, M. | https://www.embase.com/search/results?subaction=viewrecord&id=L641924170&from=export http://dx.doi.org/10.2174/1573399820666230731105704 | English |  | 10.2174/1573399820666230731105704 | did not report outcome of interest | SALMA, Duaa |
| Risk factors of young ischemic stroke in Qatar | 2007 | Clinical Neurology and Neurosurgery | | 109 | 9 | 770-773 | Khan, F. Y. | https://www.scopus.com/inward/record.uri?eid=2-s2.0-34548551443&doi=10.1016%2fj.clineuro.2007.07.006&partnerID=40&md5=5b26e52ef473f609b89cbf33b898d1b2 | | | 10.1016/j.clineuro.2007.07.006 | did not report outcome of interest | SALMA, Salma |
| Posterior circulation ischemic strokes in young adults | 2012 | Neurology | 0028-3878 | 78 | 1 |  | Laffon, M. and Suissa, L. and Mahagne, M. H. | https://www.embase.com/search/results?subaction=viewrecord&id=L70727746&from=export http://dx.doi.org/10.1212/WNL.78.1 | English | M. Laffon, St Roch Hospital, Nice, France | 10.1212/WNL.78.1 | conference abstract | SALMA, Duaa |
| Prevalence of stroke in Lebanon: A 2012 cross-sectional study | 2013 | Cerebrovascular Diseases | 1015-9770 | 35 |  | 615 | Lahoud, N. and Hosseini, H. and Salameh, P. and Saleh, N. | https://www.embase.com/search/results?subaction=viewrecord&id=L71643054&from=export http://dx.doi.org/10.1159/000353129 | English | N. Lahoud, UniversitÃ© Paris-Est, Paris, France | 10.1159/000353129 | conference abstract | Salma, Duaa |
| STROKE IN YOUNG ADULTS: A TUNISIAN COHORT | 2023 | International Journal of Stroke | 1747-4949 | 18 | 3 | 413-414 | Mimouni, E. and Jarrar, E. and Kadedi, S. and Naija, S. and Hassine, A. and Ben Amor, S. | https://www.embase.com/search/results?subaction=viewrecord&id=L642828453&from=export http://dx.doi.org/10.1177/17474930231192010 | English | E. Mimouni, University Hospital Sahloul, Neurology Departement, Sousse, Tunisia | 10.1177/17474930231192010 | conference abstract | SALMA, Duaa |
| Stroke subtypes among young Iranian adult patients | 2009 | Journal of the Neurological Sciences | 0022-510X | 285 |  | S156 | Mirbagheri, S. and Saadatnia, M. and Mollabashi, M. and Tajmirriahi, M. and Mousavi, S. A. | https://www.embase.com/search/results?subaction=viewrecord&id=L70253672&from=export | English | S. Mirbagheri, Isfahan Neurosciense Research Center, Isfahan University of Medical Sciences, Isfahan, Iran | | conference abstract | SALMA, Duaa |
| Incidence of postoperative stroke after coronary artery bypass surgery and its risk factors | 2011 | Journal of Babol University of Medical Sciences | | 13 | 3 | 75-79 | Mir-Mohammad Sadeghi, S. M. and Hadipour, M. and Molavi-e Vardanjani, H. | https://www.scopus.com/inward/record.uri?eid=2-s2.0-79957905687&partnerID=40&md5=e3767b033ba2e077a23bc20e1d3da32e | | | | did not report outcome of interest | SALMA, Duaa |
| The Burden of Stroke in Kurdistan Province, Iran from 2011 to 2017 | 2021 | Journal of Preventive Medicine and Public Health | | 54 | 2 | 103-109 | Moradi, S. and Moradi, G. and Piroozi, B. | https://www.scopus.com/inward/record.uri?eid=2-s2.0-85104212866&doi=10.3961%2fjpmph.20.335&partnerID=40&md5=b3b1c68d8177f1a1d9de18607a2f7363 | | | 10.3961/jpmph.20.335 | did not report outcome of interest | SALMA, Salma, Roaa, Duaa |
| Prevalence of cardiac risk factors in ischemic stroke in a university medical center in Tehran | 2016 | Iranian Heart Journal |  | 17 | 1 | 57-63 | Mostafavi, A. and Sekhavatfar, P. and Tabatabaei, S. A. and Khavandi, S. and Rasoulighasemlouei, S. | https://www.scopus.com/inward/record.uri?eid=2-s2.0-84977499298&partnerID=40&md5=610207cea6ef96be612ccf3317d94ed9 | | | | did not report outcome of interest | SALMA, Duaa |
| Clinical and epidemiological aspects of stroke at the University hospital of Oran Algeria | 2018 | Annals of Intensive Care | 2110-5820 | 8 | 1 |  | Mourad, G. and Djamel, A. and Houria, D. | https://www.embase.com/search/results?subaction=viewrecord&id=L620837056&from=export http://dx.doi.org/10.1186/s13613-017-0345-7 | English | G. Mourad, CHU Benaouda Benzerdje, Oran, Algeria | 10.1186/s13613-017-0345-7 | conference abstract | SALMA, Duaa |
| Prevalence of stroke among patients with chronic kidney disease, Taif, Saudi Arabia | 2023 | Saudi Medical Journal |  | 44 | 11 | 1139-1144 | Mubaraki, A. A. and Alotaibi, W. D. and Sibyani, A. K. and Alrbaiai, G. T. and Almalki, H. S. and Atallah, H. M. and Basfar, A. S. and Alqaedi, A. and Althobaiti, H. A. and Algethami, M. M. and Althobaiti, Y. A. | https://www.scopus.com/inward/record.uri?eid=2-s2.0-85176306238&doi=10.15537%2fsmj.2023.44.11.20230206&partnerID=40&md5=19e458c8ff0fa12befd1146ef523d512 | | | 10.15537/smj.2023.44.11.20230206 | did not report outcome of interest | SALMA, Duaa |
| Prevalence of stroke and associated risk factors in Lebanon: Preliminary data from the Beirut and mount Lebanon governorates | 2014 | Neuroepidemiology | 0251-5350 | 43 | 2 | 88 | Phung, T. K. T. and Atweh, S. and Chaaya, M. and Prince, M. and Waldemar, G. | https://www.embase.com/search/results?subaction=viewrecord&id=L71810966&from=export http://dx.doi.org/10.1159/000369115 | English | T.-K.-T. Phung, Danish Dementia Research Center, Department of Neurology, Rigshospitalet, University of Copenhagen, Copenhagen, Denmark | 10.1159/000369115 | conference abstract | SALMA, Duaa |
| Risk factors associated with recurrent stroke: A retrospective hospital-based study | 2019 | Journal of Acute Disease | 2221-6189 | 8 | 6 | 245-249 | Rafie, S. and Kaveyani, H. and Choghakabodi, P. M. | https://www.embase.com/search/results?subaction=viewrecord&id=L630417444&from=export http://dx.doi.org/10.4103/2221-6189.272856 | English | H. Kaveyani, Department of Neurology, Golestan Hospital, Ahvaz Jundishapur University of Medical Sciences, Ahvaz, Iran | 10.4103/2221-6189.272856 | did not report outcome of interest | SALMA, Duaa |
| Stroke epidemiology in Shiraz, Southern Iran: A hospital-based single-center retrospective longitudinal study | 2012 | European Journal of Neurology | 1351-5101 | 19 |  | 164 | Safari, A. and Safari, R. and Borhani Haghighi, A. and Sharifian, M. | https://www.embase.com/search/results?subaction=viewrecord&id=L70938874&from=export http://dx.doi.org/10.1111/j.1468-1331.2012.03888.x | English | A. Safari, Shiraz University of Medical Sciences, Shiraz, Iran | 10.1111/j.1468-1331.2012.03888.x | conference abstract | SALMA, Duaa |
| The incidence of ischemic stroke and its associated factors in young adults in Kermanshah over a seven-year period | 2019 | Journal of Kermanshah University of Medical Sciences | 2588-2570 2588-2562 | 23 | 2 |  | Sari-Aslani, P. and Sultanabadi, R. and Hosseini, F. and Mohammadi, H. | https://www.embase.com/search/results?subaction=viewrecord&id=L2002242207&from=export http://dx.doi.org/10.5812/jkums.90139 | English | H. Mohammadi, Department of Neurology, School of Medicine, Kermanshah University of Medical Sciences, Kermanshah, Iran | 10.5812/jkums.90139 | can't be accessed | SALMA, Roaa, Duaa |
| Cardiovascular Disease in Iran in the Last 40 Years: Prevalence, Mortality, Morbidity, Challenges and Strategies for Cardiovascular Prevention | 2019 | Arch Iran Med | 1029-2977 | 22 | 4 | 204-210 | Sarrafzadegan, N. and Mohammmadifard, N. | | eng | Isfahan Cardiovascular Research Center, Cardiovascular Research Institute, Isfahan University of Medical Sciences, Isfahan, Iran. Hypertension Research Center, Cardiovascular Research Institute, Isfahan University of Medical Sciences, Isfahan, Iran. | | did not report outcome of interest | SALMA, Duaa |
| Burden of stroke in North Africa and Middle East, 1990 to 2019: a systematic analysis for the global burden of disease study 2019 | 2022 | BMC Neurology |  | 22 | 1 |  | Shahbandi, A. and Shobeiri, P. and Azadnajafabad, S. and Saeedi Moghaddam, S. and Sharifnejad Tehrani, Y. and Ebrahimi, N. and Rezaei, N. and Rashidi, M. M. and Ghamari, S. H. and Abbasi-Kangevari, M. and Koolaji, S. and Haghshenas, R. and Rezaei, N. and Larijani, B. and Farzadfar, F. | https://www.scopus.com/inward/record.uri?eid=2-s2.0-85134901010&doi=10.1186%2fs12883-022-02793-0&partnerID=40&md5=658dbd9f3306979e969766590892d3e6 | | | 10.1186/s12883-022-02793-0 | did not report outcome of interest | SALMA, Duaa |
| Epidemiology of stroke in the central area of Iran; a populationbased study | 2020 | International Journal of Stroke | 1747-4949 | 15 | 1 | 400-401 | Sharifipour, E. and Aghaali, M. and Yousofi, S. | https://www.embase.com/search/results?subaction=viewrecord&id=L634009672&from=export http://dx.doi.org/10.1177/1747493020963387 | English | E. Sharifipour, Neurosciences Research Center, Qom University of Medical Sciences, Neurology, Iran | 10.1177/1747493020963387 | conference abstract | SALMA, Duaa |
| Prevalence of cardiovascular diseases and vascular risk factors in patients with history of epilepsy/seizures | 2020 | Neurology | 1526-632X | 94 | 15 |  | Yassin, A. and Al-Mistarehi, A. H. and El-Salem, K. and Almegdadi, A. and Ennab, M. and Ahmad, M. and Kareem, A. and Tashtoush, A. and Almasri, S. and Kana'an, M. and Dabour, B. and Aqaileh, S. | https://www.embase.com/search/results?subaction=viewrecord&id=L633066889&from=export | English | A. Yassin, Jordan University of Science and Tech | | conference abstract | Roaa, Duaa |
| Global Burden of Ischemic Stroke in Young Adults in 204 Countries and Territories | 2023 | Neurology |  | 100 | 4 | E422-E434 | Zhang, R. and Liu, H. and Pu, L. and Zhao, T. and Zhang, S. and Han, K. and Han, L. | https://www.scopus.com/inward/record.uri?eid=2-s2.0-85147045836&doi=10.1212%2fWNL.0000000000201467&partnerID=40&md5=1d0512c43d83ab962efb0b1e1cf41c1e | | | 10.1212/WNL.0000000000201467 | full text not found | SALMA, Duaa |
| Global, regional, and national burden of ischemic stroke, 1990-2021: an analysis of data from the global burden of disease study 2021 | 2024 | EClinicalMedicine | 2589-5370 | 75 |  | 102758 | Li, X. Y. and Kong, X. M. and Yang, C. H. and Cheng, Z. F. and Lv, J. J. and Guo, H. and Liu, X. H. | | eng | Department of Neurology, Shanghai Putuo People's Hospital, School of Medicine, Tongji University, Shanghai, China. Department of Plastic and Reconstructive Surgery, Shanghai Ninth People's Hospital, Shanghai Jiao Tong University, Shanghai, China. Department of Vascular Surgery, Shanghai Putuo People's Hospital, School of Medicine, Tongji University, Shanghai, China. Department of Neurosurgery, Shanghai Ninth People's Hospital, Shanghai Jiao Tong University, Shanghai, PR China. Department of Gynecology and Obstetrics, Tongji Hospital, Tongji University School of Medicine, Shanghai, China. | 10.1016/j.eclinm.2024.102758 | wrong population | Duaa, Salma |
| EPIDEMIOLOGY AND ASSOCIATED RISK FACTORS OF ISCHEMIC STROKE TREATED AT THE UNIVERSITY HOSPITAL CENTER OF TLEMCEN (ALGERIA) | 2024 | Journal of Hypertension | 0263-6352 | 42 |  | e308-e308 | Abdrebbi, Samira Benbekhti | https://search.ebscohost.com/login.aspx?direct=true&db=cul&AN=177434430&site=ehost-live&scope=site&authtype=sso&custid=s3704754 | | Faculty of medicine | 10.1097/01.hjh.0001022708.19992.be | Conference paper | Duaa, Salma |
| The burden of neurological conditions in north Africa and the Middle East, 1990â€“2019: a systematic analysis of the Global Burden of Disease Study 2019 | 2024 | The Lancet Global Health | | 12 | 6 | e960-e982 | Avan, A. and Feigin, V. L. and Bennett, D. A. and Steinmetz, J. D. and Hachinski, V. and Stranges, S. and Owolabi, M. O. and Aali, A. and Abbasi-Kangevari, M. and Abbasi-Kangevari, Z. and Abd-Allah, F. and Abdollahzade, S. and Abidi, H. and Abolhassani, H. and Abualhasan, A. and Abu-Gharbieh, E. and Abu-Rmeileh, N. M. and Abu-Zaid, A. and Ahmad, A. and Ahmadi, S. and Ahmed, L. A. and Ajami, M. and Al Hamad, H. and Alanezi, F. M. and Alanzi, T. M. and Alimohamadi, Y. and Aljunid, S. M. and Al-Raddadi, R. M. and Amiri, S. and Arabloo, J. and Arulappan, J. and Arumugam, A. and Asadi-Pooya, A. A. and Athar, M. and Athari, S. S. and Atout, M. M. W. and Azadnajafabad, S. and Azangou-Khyavy, M. and Azari Jafari, A. and Azzam, A. Y. and Baghcheghi, N. and Bagherieh, S. and Baltatu, O. C. and Bazmandegan, G. and Bhojaraja, V. S. and Bijani, A. and Bitaraf, S. and Calina, D. and Darwish, A. H. and Djalalinia, S. and Doheim, M. F. and Dorostkar, F. and Eini, E. and El Nahas, N. and El Sayed, I. and Elhadi, M. and Elmonem, M. A. and Eskandarieh, S. and Faghani, S. and Fallahzadeh, A. and Farahmand, M. and Ghafourifard, M. and Ghamari, S. H. and Gholami, A. and Ghozy, S. and Goleij, P. and Hadei, M. and Hafezi-Nejad, N. and Haj-Mirzaian, A. and Halwani, R. and Hamidi, S. and Hasaballah, A. I. and Hassan, A. and Hedna, K. and Hegazy, M. I. and Heidari-Soureshjani, R. and Hosseini, M. S. and Hoveidamanesh, S. and Jahrami, H. and Jamshidi, E. and Javaheri, T. and Jayapal, S. K. and Kalankesh, L. R. and Kalhor, R. and Kamiab, Z. and Keykhaei, M. and Khader, Y. S. and Khan, M. and Khan, M. A. and Khayat Kashani, H. R. and Khosravi, A. and Kompani, F. and Koohestani, H. R. and Larijani, B. and Lasrado, S. and Magdy Abd El Razek, M. and Malekpour, M. R. and Malik, A. A. and Mansournia, M. A. and Mardi, P. and Maroufi, S. F. and Masoudi, S. and Mayeli, M. and Mehrabi Nasab, E. and Menezes, R. G. and Mirmoeeni, S. and Mirza-Aghazadeh-Attari, M. and Mobarakabadi, M. and Mohammadi, E. and Mohammadi, S. and Mohan, S. and Mokdad, A. H. and Momtazmanesh, S. and Montazeri, F. and Moradi Sarabi, M. and Moraga, P. and Morovatdar, N. and Motaghinejad, M. and Naghavi, M. and Natto, Z. S. and Nejadghaderi, S. A. and Noroozi, N. and Okati-Aliabad, H. and Pazoki Toroudi, H. and Perna, S. and Piradov, M. A. and Pourahmadi, M. and Rafiei, A. and Rahimi-Movaghar, V. and Rahmani, A. M. and Rahmani, S. and Rahmanian, V. and Rajabpour-Sanati, A. and Rao, C. R. and Rashidi, M. M. and Rawassizadeh, R. and Razeghian-Jahromi, I. and Redwan, E. M. M. and Rezaee, M. and Rezaei, N. and Rezaei, N. and Rezaei, N. and Rezaeian, M. and Rikhtegar, R. and Saad, A. M. A. and Saddik, B. and Sadeghi, M. and Sadeghian, S. and Saeedi Moghaddam, S. | https://www.scopus.com/inward/record.uri?eid=2-s2.0-85193773695&doi=10.1016%2fS2214-109X%2824%2900093-7&partnerID=40&md5=67167df8e6082b6aa46d8144e3321420 | | | 10.1016/S2214-109X(24)00093-7 | did not report outcome of interest | Duaa, Salma |
| Epidemiological profile of stroke in Qatar: Insights from a seven-year observational study | 2024 | J Clin Neurosci | 0967-5868 | 123 |  | 30-35 | Bhutta, Z. A. and Akhtar, N. and Pathan, S. A. and Castren, M. and Harris, T. and Ganesan, G. S. and Kamran, S. and Thomas, S. H. and Cameron, P. A. and Azad, A. M. and Puolakka, T. | | eng | Department of Emergency Medicine, Hamad Medical Corporation, Doha, Qatar; Department of Emergency Medicine and Services, Helsinki University Hospital and University of Helsinki, Helsinki, Finland. Electronic address: zain.bhutta@helsinki.fi. Department of Neurology, Neuroscience Institute, Hamad Medical Corporation, Doha, Qatar. Electronic address: nakhtar@hamad.qa. Department of Emergency Medicine, Hamad Medical Corporation, Doha, Qatar; Blizard Institute of Barts & The London School of Medicine, Queen Mary University of London, UK; School of Public Health and Preventive Medicine, Monash University, Melbourne, Australia. Electronic address: SPathan@hamad.qa. Department of Emergency Medicine and Services, Helsinki University Hospital and University of Helsinki, Helsinki, Finland. Electronic address: maaret.castren@hus.fi. Blizard Institute of Barts & The London School of Medicine, Queen Mary University of London, UK. Electronic address: t.harris@qmul.ac.uk. Department of Emergency Medicine, Hamad Medical Corporation, Doha, Qatar. Electronic address: GGanesan@hamad.qa. Department of Neurology, Neuroscience Institute, Hamad Medical Corporation, Doha, Qatar. Electronic address: skamranmd@hotmail.com. Blizard Institute of Barts & The London School of Medicine, Queen Mary University of London, UK; Department of Emergency Medicine, Beth Israel Deaconess Medical Center and Harvard Medical School, Boston, USA. Electronic address: sthomasmd@gmail.com. The Alfred Hospital, Emergency and Trauma Centre, School of Public Health and Preventive Medicine, Monash University, Melbourne, Australia. Electronic address: peter.cameron@monash.edu. Department of Emergency Medicine, Hamad Medical Corporation, Doha, Qatar. Electronic address: Aazad@hamad.qa. Department of Emergency Medicine and Services, Helsinki University Hospital and University of Helsinki, Helsinki, Finland. Electronic address: Tuukka.Puolakka@hus.fi. | 10.1016/j.jocn.2024.03.014 | did not report outcome of interest | Duaa, Salma |
| Incidence, clinical features, and outcomes of posterior circulation ischemic stroke: insights from a large multiethnic stroke database | 2024 | Front Neurol | 1664-2295 (Print) 1664-2295 | 15 |  | 1302298 | Imam, Y. Z. and Chandra, P. and Singh, R. and Hakeem, I. and Al Sirhan, S. and Kotob, M. and Akhtar, N. and Kamran, S. and Al Jerdi, S. and Muhammad, A. and Haroon, K. H. and Hussain, S. and Perkins, J. D. and Elalamy, O. and Alhatou, M. and Ali, L. and Abdelmoneim, M. S. and Joseph, S. and Morgan, D. and Uy, R. T. and Bhutta, Z. and Azad, A. and Ayyad, A. and Elsotouhy, A. and Own, A. and Deleu, D. | | eng | Neuroscience Institute, Hamad Medical Corporation, Doha, Qatar. Weill Cornell Medicine-Qatar, Doha, Qatar. College of Medicine, Qatar University, Doha, Qatar. Statistics, Medical Research Center, Hamad Medical Corporation, Doha, Qatar. Cardiology Research Center, Hamad Medical Corporation, Doha, Qatar. Department of Emergency Medicine, Hamad Medical Corporation, Doha, Qatar. | 10.3389/fneur.2024.1302298 | wrong population | Duaa, Salma |
| Spiral strapping for improving upper extremity motor functions in individuals with stroke: A randomized controlled trial | 2024 | Prosthetics and orthotics international | 1746-1553 |  |  |  | Abd El-Kafy, E. M. and Alayat, M. S. and Alwhaibi, R. M. and Basuodan, R. M. | https://www.embase.com/search/results?subaction=viewrecord&id=L643177881&from=export http://dx.doi.org/10.1097/PXR.0000000000000325 | English |  | 10.1097/PXR.0000000000000325 | wrong study design | Duaa, Salma |
| TICA-CLOP STUDY: Ticagrelor Versus Clopidogrel in Acute Moderate and Moderate-to-Severe Ischemic Stroke, a Randomized Controlled Multi-Center Trial | 2024 | CNS Drugs | 1172-7047 |  |  |  | Ahmed, S. R. and Nahas, N. E. and Khalil, M. F. E. and Elbassiouny, A. and Almoataz, M. A. and Omar, T. Y. and Daabis, A. M. A. and Refat, H. M. and Ebied, Aamk and Hassan, A. M. and Mohamed, D. M. A. and Ismaiel, M. and Zeinhom, M. G. | | eng | Neurology Department, Faculty of Medicine, Kafr el-sheikh University, Elgeish Street, Kafr el-sheikh, Egypt. Neurology Department, Faculty of Medicine, Ain shams University, Al Khalifa Elmamon St., Cairo, Egypt. Neurology Department, Phoenix Hospital, Shabia 10, Mussafah, Abu Dhabi, United Arab Emirates. Neurology Department, Saudi German Hospital, Sharjah, United Arab Emirates. Neurology Department, Burjeel Medical Centers, Abu Dhabi, United Arab Emirates. Neurology Department, Burjeel Royal Hospital, Al Ain, United Arab Emirates. Neurology Department, Faculty of Medicine, Zagazig University, 2 elgeish Et, Zagazig, Egypt. Neurology Department, Medeor Hospital, Abu Dhabi, UAE. Neurology Department, Al Dhafra Hospitals, Abu Dhabi, UAE. Neurology Department, Al-Sahel Teaching Hospital, 12 Shubra st Cairo, Cairo, Egypt. Neurology Department, Faculty of Medicine, Kafr el-sheikh University, Elgeish Street, Kafr el-sheikh, Egypt. mohamed_gomaa@med.kfs.edu.eg. | 10.1007/s40263-024-01127-7 | wrong study design | Duaa, Salma |
| Stroke and high-risk TIA outcomes with reduction of treatment duration when treatment initiated in emergency rooms (SHORTER-study) | 2024 | Int J Stroke | 1747-4930 | 19 | 7 | 830-834 | Alhazzani, A. and Alajlan, F. S. and Alkhathaami, A. M. and Al-Senani, F. M. and Muayqil, T. A. and Alghamdi, S. A. and AlKawi, A. and AlZahrani, S. and Bakheet, M. and Aljohani, M. and Taher, N. and Almutairi, A. and AlQarni, M. and Alsalman, S. and Alqahtani, S. A. and Almansour, N. and Abukhamsin, L. and Mouminah, A. and Almodarra, N. and Mohamed, G. and Almodhy, M. and Albogumi, E. and Alzawahmah, M. and Alreshaid, A. and Akhtar, N. and Hussain, M. S. and Albers, G. W. and Shuaib, A. | | eng | King Faisal Specialist Hospital & Research Center, Riyadh, Saudi Arabia. College of Medicine, King Saud Bin Abdulaziz University for Health Sciences, Riyadh, Saudi Arabia. National Neuroscience Institute, King Fahad Medical City, Riyadh, Saudi Arabia. Neurology Unit, Department of Medicine, College of Medicine, King Saud University, Riyadh, Saudi Arabia. King Faisal Specialist Hospital & Research Center, Jeddah, Saudi Arabia. King Fahad General Hospital, Ministry of Health, Jeddah, Saudi Arabia. King Abdullah Medical City, Mecca, Saudi Arabia. King Salman ibn Abdulaziz Medical City, Madinah, Saudi Arabia. Prince Sultan Military Medical City, Riyadh, Saudi Arabia. King Fahad Hospital of the University, Imam Abdulrahman Bin Faisal University, Dammam, Saudi Arabia. King Fahad Hospital Hofuf, Al-Ahsa, Riyadh, Saudi Arabia. College of Medicine, King Khalid University, Abha, Saudi Arabia. King Saud Medical City, Riyadh, Saudi Arabia. Dammam Medical Complex, Dammam, Saudi Arabia. King Abdullah Medical Complex, Jeddah, Saudi Arabia. Hamad Medical Corporation, Doha, Qatar. Cleveland Clinic, Cleveland, OH, USA. Stanford University, Stanford, CA, USA. University of Alberta, Edmonton, AB, Canada. | 10.1177/17474930241237120 | wrong study design | Duaa, Salma |
| Correction: Repetitive peripheral magnetic stimulation for improving upper limb function in post-stroke hemiparesis (Egyptian Rheumatology and Rehabilitation, (2023), 50, 1, (35), 10.1186/s43166-023-00204-x) | 2024 | Egyptian Rheumatology and Rehabilitation | | 51 | 1 |  | Fawaz, S. I. and Izumi, S. I. and Zaki, A. S. and Eldiasty, S. E. and Saadawy, A. and Saber, H. G. E. and Gadallah, M. F. and Labib, H. S. | https://www.scopus.com/inward/record.uri?eid=2-s2.0-85195360981&doi=10.1186%2fs43166-024-00247-8&partnerID=40&md5=67b6ddf653e571c9a0a3f39ab4131a0a | | | 10.1186/s43166-024-00247-8 | wrong study design | Salma, Roaa |
| RandomizÃ¡lt, kontrollÃ¡lt klinikai vizsgÃ¡latok nem traumÃ¡s agyÃ¡llomÃ¡nyi vÃ©rzÃ©sben | 2024 | Lege Artis Medicine (LAM) | 0866-4811 | 34 | 7 | 346-353 | HornyÃK, Csilla and Bereczki, DÃNiel | https://search.ebscohost.com/login.aspx?direct=true&db=cul&AN=179411284&site=ehost-live&scope=site&authtype=sso&custid=s3704754 | | Semmelweis Egyetem, NeurolÃ³giai Klinika | 10.33616/lam.34.0346 | wrong study design | Duaa, Salma |
| Investigation of the Effectiveness of Doxycycline on the Improvement of Oxidative Stress Biomarkers in Patients with Ischemic Stroke | 2024 | Avicenna Journal of Clinical Medicine | | 30 | 4 | 194-201 | Khazaei, M. and Valdbeighi, M. and Khazaei, S. and Mirmoeini, E. A. and Mehri, F. | https://www.scopus.com/inward/record.uri?eid=2-s2.0-85188921629&doi=10.32592%2fajcm.30.4.194&partnerID=40&md5=adb9968e7bd9b3655d095c11ef5e4789 | | | 10.32592/ajcm.30.4.194 | wrong study design | Duaa, Salma |
| The Effect of Intravenous Alteplase on Patients with Acute Ischemic Stroke: A Clinical Trial Study in a Specialty Hospital in Ilam | 2024 | Journal of Basic Research in Medical Sciences | 2383-0506 | 11 | 2 | 62-69 | MohamadYari, Milad and Rahmatian, Aryoobarzan and Azizi, Monireh and Asadollahi, Khairollah | https://search.ebscohost.com/login.aspx?direct=true&db=cul&AN=177643465&site=ehost-live&scope=site&authtype=sso&custid=s3704754 | | Student research committee, Ilam university of Medical Sciences, Ilam, Iran Department of Neurology, Faculty of Medicine, Ilam University of Medical Sciences, Ilam, Iran Department of Anatomy, Faculty of Medicine, Ilam University of Medical Sciences, Ilam, Iran Department of Social Medicine, Faculty of Medicine, Ilam University of Medical Sciences, Ilam, Iran | | wrong study design | Duaa, Salma |
| Effect of Colchicine for Prevention of Recurrent Stroke in Ischemic Stroke Patients with Atrial Fibrillation: A Randomized Double-blinded Placebo-- controlled Trial | 2024 | Rev Recent Clin Trials | 1574-8871 |  |  |  | Molaeimanesh, Z. and Kashipazha, D. and Shalilahmadi, D. and Shamsaei, G. and Mohammadi, S. | | eng | Department of Neurology, Faculty of Medicine, Ahvaz Jundishapur University of Medical Sciences, Ahvaz, Iran. | 10.2174/0115748871325292240904060109 | wrong study design | Duaa, Salma |
| Impact of Hope on Stroke Patients Receiving a Spiritual Care Program in Iran: A Randomized Controlled Trial | 2024 | J Relig Health | 0022-4197 (Print) 0022-4197 | 63 | 1 | 356-369 | Oshvandi, K. and Torabi, M. and Khazaei, M. and Khazaei, S. and Yousofvand, V. | | eng | Department of Medical Surgical Nursing, Mother and Child Care Research Center, School of Nursing and Midwifery, Hamadan University of Medical Sciences, Hamadan, Iran. Chronic Diseases (Home Care) Research Centre, Malayer School of Nursing, Hamadan University of Medical Sciences, Hamadan, Iran. Besat Educational and Medical Center, School of Medicine, Hamadan University of Medical Sciences, Hamadan, Iran. Research Center for Health Sciences, Hamadan University of Medical Sciences, Hamadan, Iran. Student Research Committee, Hamadan University of Medical Sciences, Hamadan, Iran. v.yousofvand@gmail.com. | 10.1007/s10943-022-01696-1 | wrong study design | Duaa, Salma |
| Clinical trial of the effects of postbiotic supplementation on inflammation, oxidative stress, and clinical outcomes in patients with CVA | 2024 | Sci Rep | 2045-2322 | 14 | 1 | 24021 | Rahimi, A. and Qaisar, S. A. and Janeh, T. and Karimpour, H. and Darbandi, M. and Moludi, J. | | eng | Clinical Research Development Center, Imam Reza Hospital, Kermanshah University of Medical Sciences, Kermanshah, Iran. Chemistry Department, College of Education, University of Garmian, Sulimmania, Iraq. Research Center for Environmental Determinants of Health (RCEDH), Health Institute, Kermanshah University of Medical Sciences, Kermanshah, Iran. Student Research Committee, Kermanshah University of Medical Sciences, Kermanshah, Iran. Clinical Research Development Center, Imam Reza Hospital, Kermanshah University of Medical Sciences, Kermanshah, Iran. jmoludi@yahoo.com. School of Nutrition Sciences and Food Technology, Kermanshah University of Medical Sciences, Kermanshah, 5166614711, 00989399516760, Iran. jmoludi@yahoo.com. | 10.1038/s41598-024-76153-y | wrong study design | Salma, Roaa |
| Ticagrelor Versus Clopidogrel in Acute Large-Vessel Ischemic Stroke: A Randomized Controlled Single-Blinded Trial | 2024 | CNS Drugs | 1172-7047 (Print) 1172-7047 | 38 | 5 | 387-398 | Zeinhom, M. G. and Elbassiouny, A. and Mohamed, A. M. and Ahmed, S. R. | | eng | Neurology Department, Faculty of Medicine, Kafr El-Sheikh University, Elgeish St, Kafr El-Sheikh, Egypt. mohamed_gomaa@med.kfs.edu.eg. Neurology Department, Faculty of Medicine, Ain Shams University, Al Khalifa Elmamon St, Cairo, Egypt. Neurology Department, Damietta Hospital, Algomhoria St, Damietta, Egypt. Neurology Department, Faculty of Medicine, Kafr El-Sheikh University, Elgeish St, Kafr El-Sheikh, Egypt. | 10.1007/s40263-024-01080-5 | wrong study design | Duaa, Salma |
| An Uncommon Coexistence of Ischemic Stroke in a Multiple Sclerosis Patient With Duplicated Superior Cerebellar Artery: A Case Report From Saudi Arabia | 2024 | Cureus | 2168-8184 (Print) 2168-8184 | 16 | 5 | e60218 | AlQahtani, B. G. and Kajtazi, N. and Aljaidi, H. K. and Alhatlany, K. | | eng | Neurology Department, Prince Sultan Military Medical City, Riyadh, SAU. | 10.7759/cureus.60218 | wrong study design | Salma, Roaa |
| Should the Left Atrial Appendage Closure (LAAC) Technique Be the Main Form of Stroke Prevention in Patients With Long-Standing Persistent or Permanent Atrial Fibrillation? | 2024 | Cureus | 2168-8184 (Print) 2168-8184 | 16 | 2 | e54256 | Amalathasan, T. and Nagaratnam, P. A. and El Dirani, M. and Nagaratnam, J. M. and Kholoki, S. | | eng | Internal Medicine, All Saints University School of Medicine, Chicago, USA. General Medicine, Bond University, Gold Coast, AUS. Internal Medicine, Saint James School of Medicine, Chicago, USA. Internal Medicine, Avalon University School of Medicine, Phoenix, USA. Internal Medicine, La Grange Memorial Hospital, Chicago, USA. | 10.7759/cureus.54256 | wrong population | SALMA, Duaa |
| Multiple cerebral hydatid cysts: A rare case report | 2024 | Interdisciplinary Neurosurgery: Advanced Techniques and Case Management | 2214-7519 | 36 |  |  | Armanfar, M. and Motavallihaghi, S. and Heidari, S. and Ghasemikhah, R. | https://www.embase.com/search/results?subaction=viewrecord&id=L2028616059&from=export http://dx.doi.org/10.1016/j.inat.2023.101878 | English | R. Ghasemikhah, Department of Parasitology and Mycology, School of Medicine, Arak University of Medical Sciences, Arak, Iran | 10.1016/j.inat.2023.101878 | wrong poulation | SALMA, Duaa, Roaa |
| Hereditary TTP in pregnant saudi lady with family history of TTP | 2024 | Clin. Med. J. R. Coll. Phys. Lond. | 1473-4893 1470-2118 | 24 |  |  | Elimam, A. and Elimam, A. and Khalil, K. | https://www.embase.com/search/results?subaction=viewrecord&id=L2033327565&from=export http://dx.doi.org/10.1016/j.clinme.2024.100081 | English |  | 10.1016/j.clinme.2024.100081 | wrong population | SALMA, Roaa |
| A CASE REPORT OF AUTOIMMUNE ENCEPHALITIS IN AN ELDERLY MALE PATIENT WITH SUDDEN-ONSET ALTERED MENTAL STATUS MIMICKING STROKE | 2024 | Chest | 1931-3543 0012-3692 | 166 |  | A2415-A2416 | Khan, I. and Afroze, T. and Hafiz, S. and Mirembe, L. and Basbayraktar, B. | https://www.embase.com/search/results?subaction=viewrecord&id=L2034599860&from=export http://dx.doi.org/10.1016/j.chest.2024.06.1477 | English |  | 10.1016/j.chest.2024.06.1477 | wrong population | Salma, Roaa |
| Takayasu arteritis presenting with large cerebral infarction in a 39-year-old Syrian woman: a case report | 2024 | Ann Med Surg (Lond) | 2049-0801 (Print) 2049-0801 | 86 | 6 | 3762-3765 | Muhammad, T. and Hamdan, Z. and Ebrahim, M. and Ali, B. and Hamdan, J. | | eng | Department of Rheumatology. Medical Student at Faculty of Medicine, Damascus University. Department of Radiology, Tishreen Military Hospital. Department of Otolaryngology, Military Hospital 601, Damascus, Syria. | 10.1097/ms9.0000000000002098 | wrong study design | Salma, Duaa |
