# Supplementary figures and images for: Prevalence of stroke in young adults in the Middle East and North Africa Region: A systematic review and meta-analysis
Source: PLOS Glob Public Health. 2025 Oct 6;5(10):e0004666. doi: 10.1371/journal.pgph.0004666 (PMC12500109; doi:10.1371/journal.pgph.0004666)

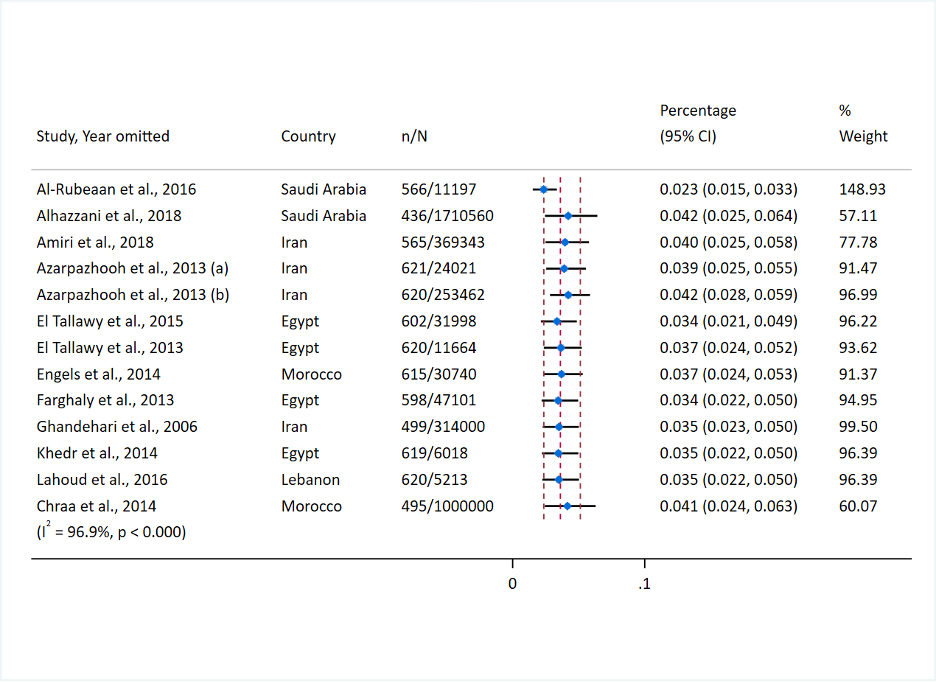

Supplement: S1 Fig — (TIF) [file pgph.0004666.s006.tif]

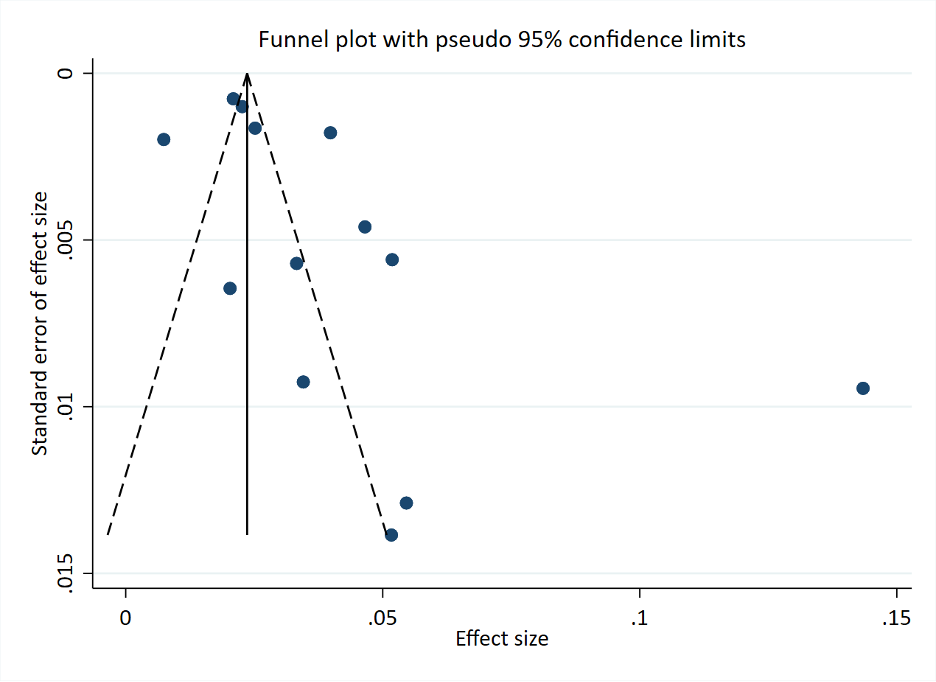

Supplement: S2 Fig — (TIF) [file pgph.0004666.s007.tif]

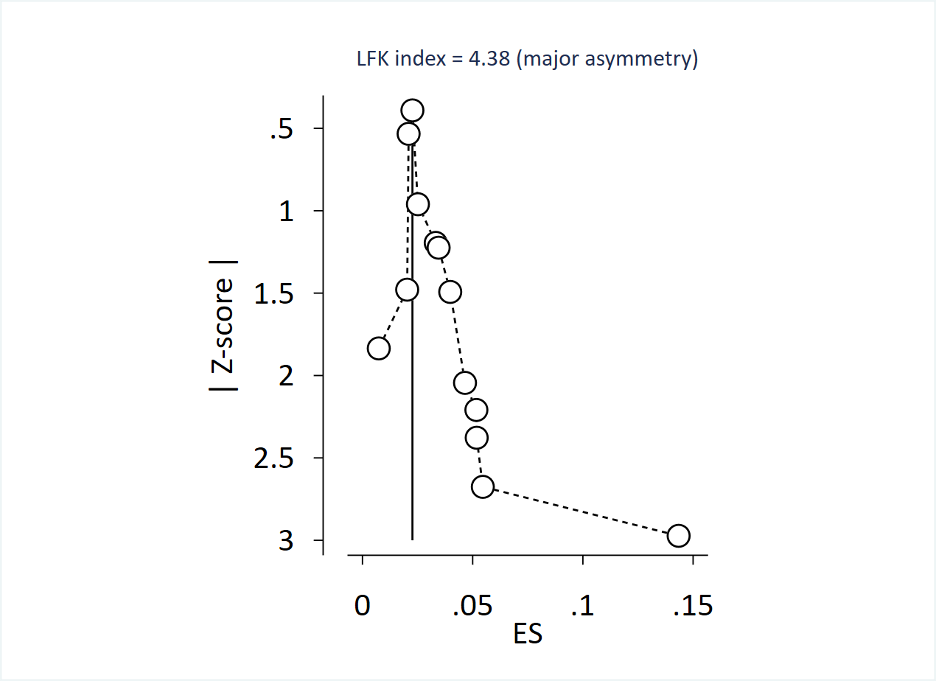

Supplement: S3 Fig — (TIF) [file pgph.0004666.s008.tif]

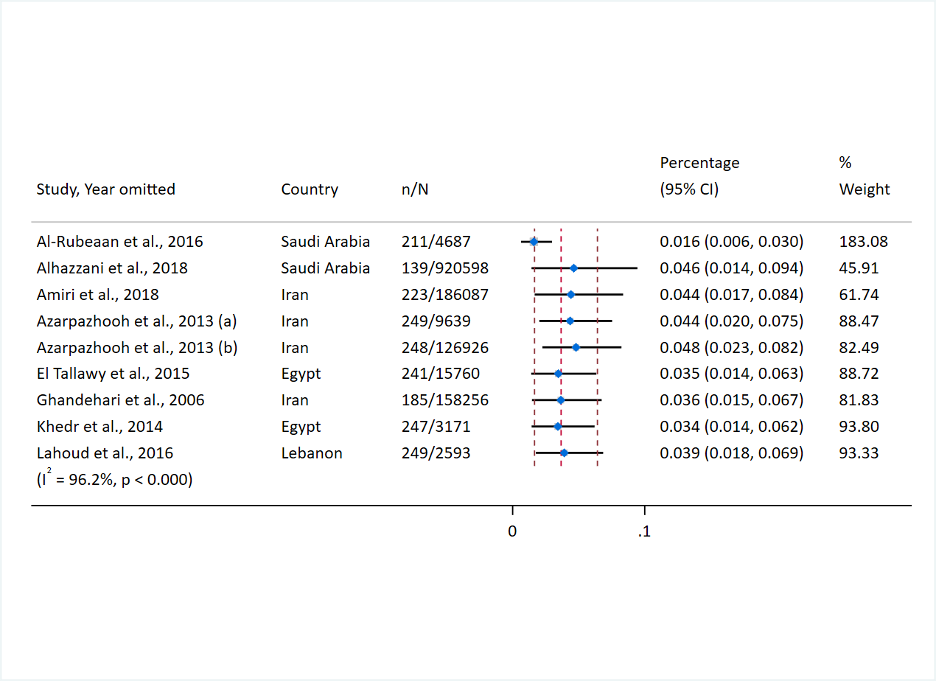

Supplement: S4 Fig — (TIF) [file pgph.0004666.s009.tif]

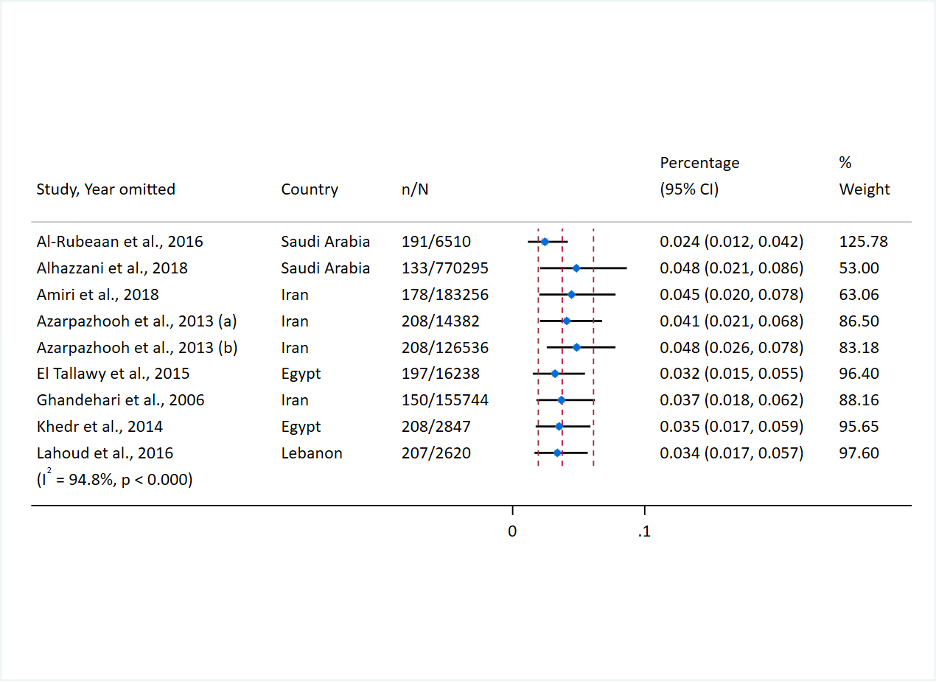

Supplement: S5 Fig — (TIF) [file pgph.0004666.s010.tif]

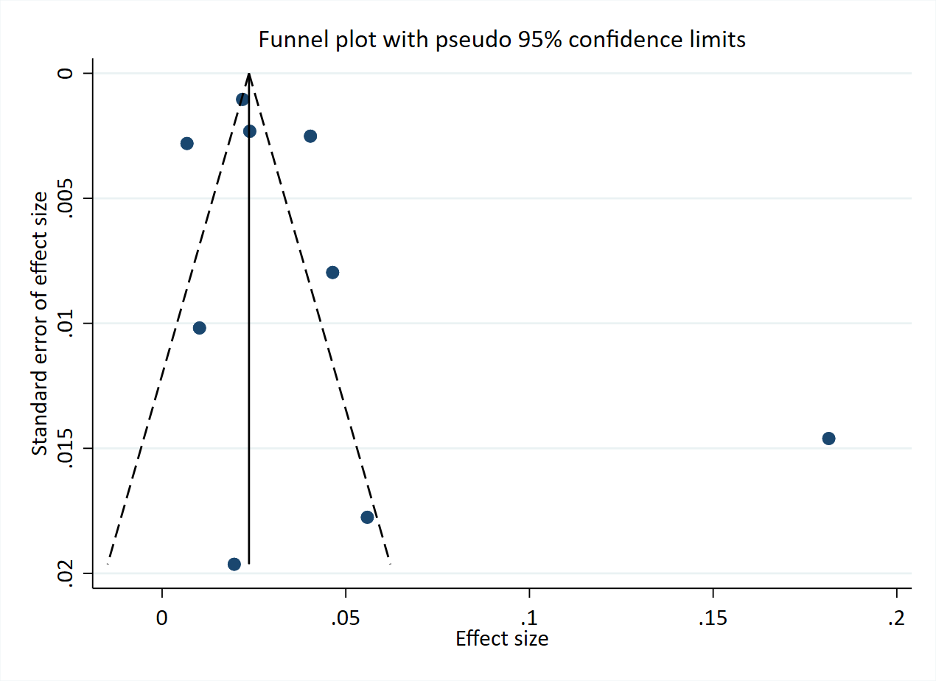

Supplement: S6 Fig — (TIF) [file pgph.0004666.s011.tif]

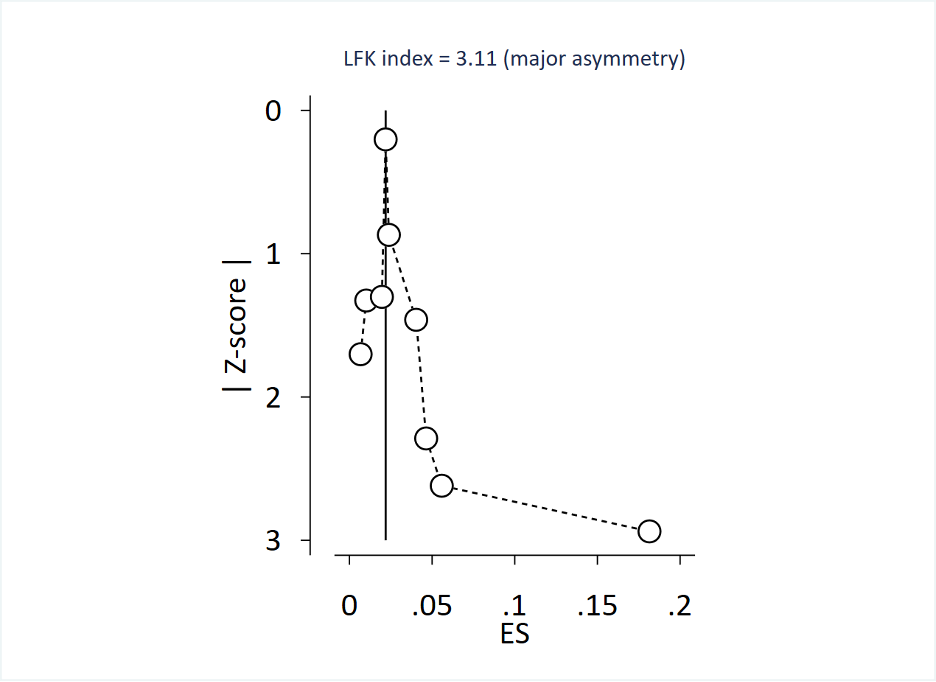

Supplement: S7 Fig — (TIF) [file pgph.0004666.s012.tif]

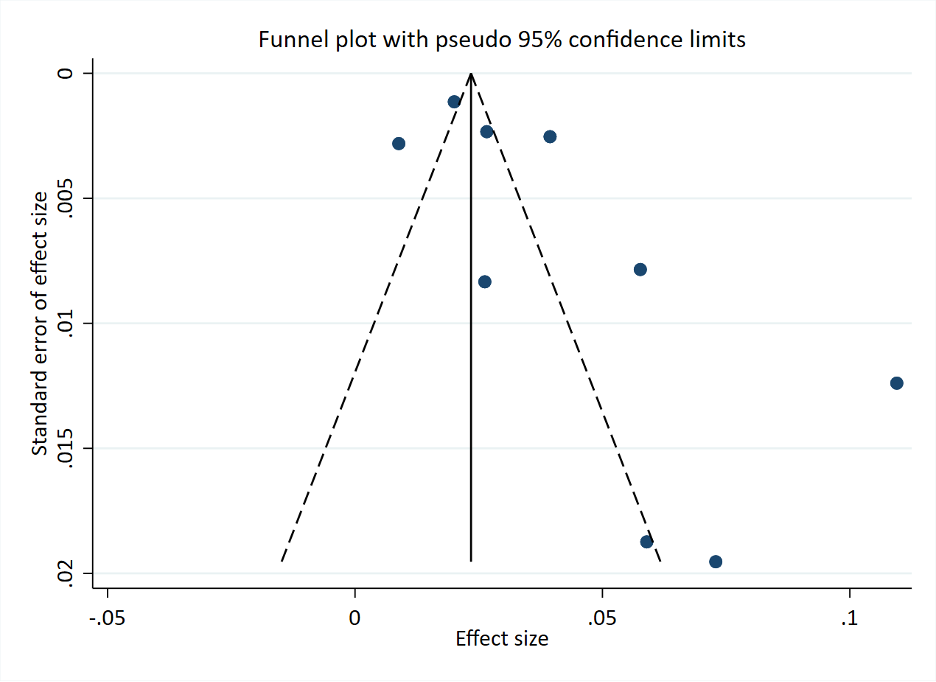

Supplement: S8 Fig — (TIF) [file pgph.0004666.s013.tif]

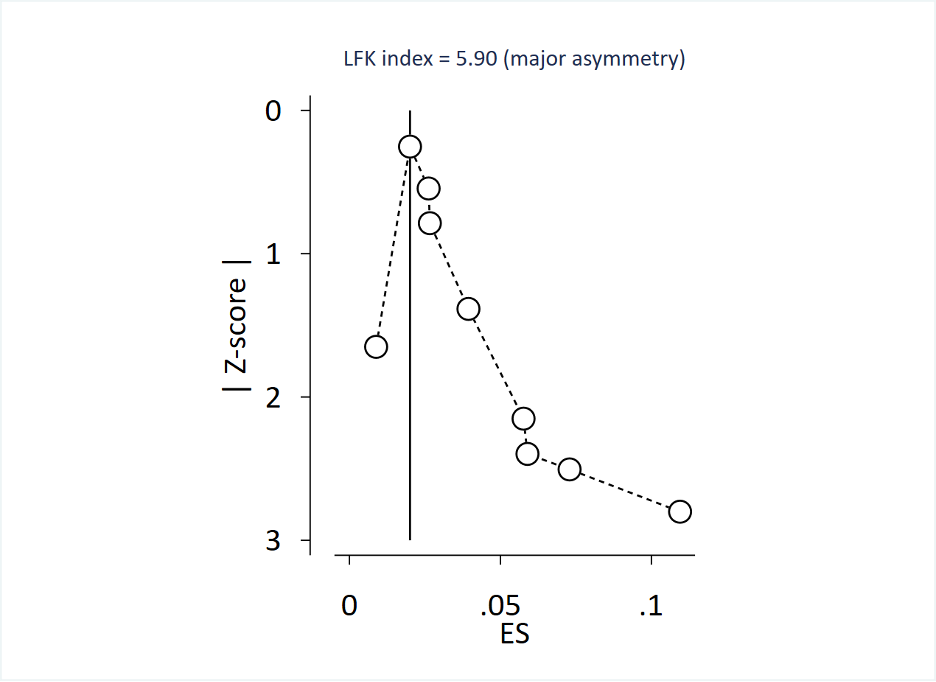

Supplement: S9 Fig — (TIF) [file pgph.0004666.s014.tif]

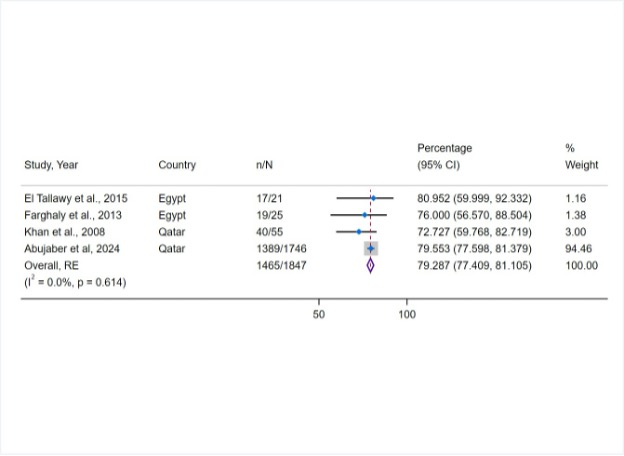

Supplement: S10 Fig — (TIF) [file pgph.0004666.s015.tif]

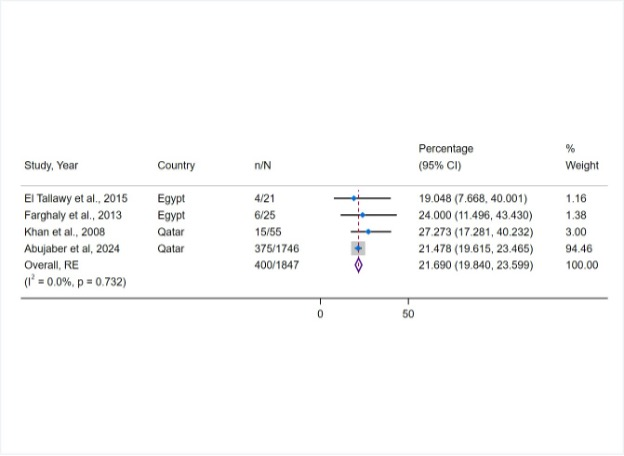

Supplement: S11 Fig — (TIF) [file pgph.0004666.s016.tif]

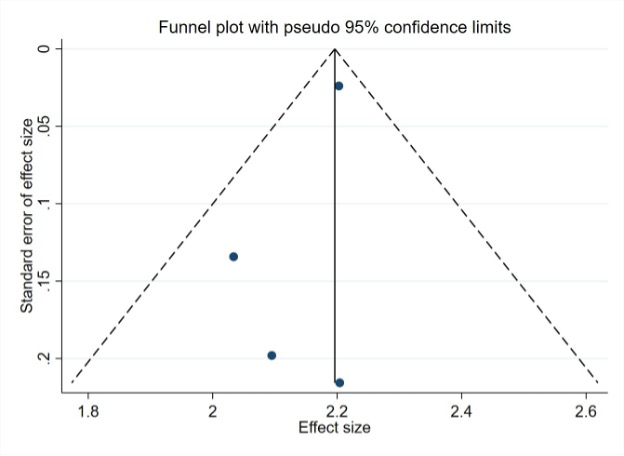

Supplement: S12 Fig — (TIF) [file pgph.0004666.s017.tif]

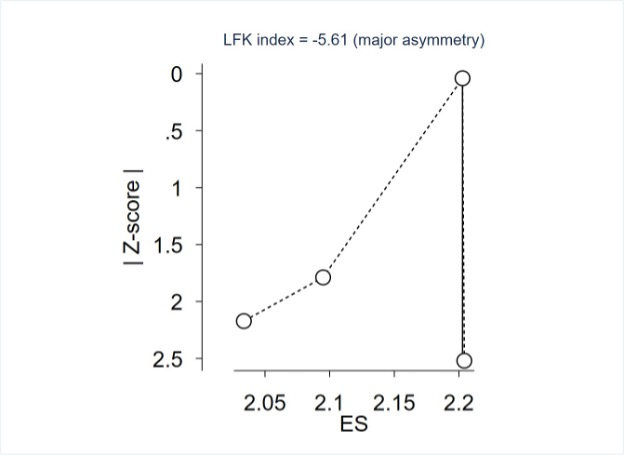

Supplement: S13 Fig — (TIF) [file pgph.0004666.s018.tif]

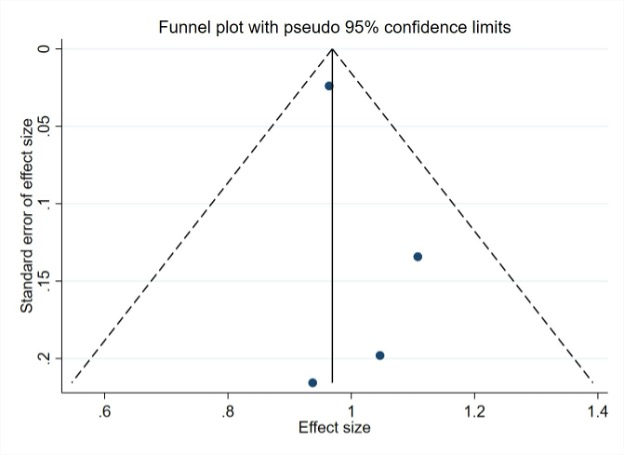

Supplement: S14 Fig — (TIF) [file pgph.0004666.s019.tif]

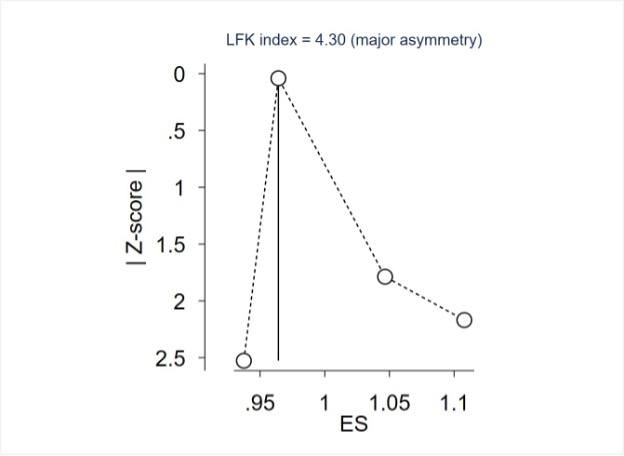

Supplement: S15 Fig — (TIF) [file pgph.0004666.s020.tif]

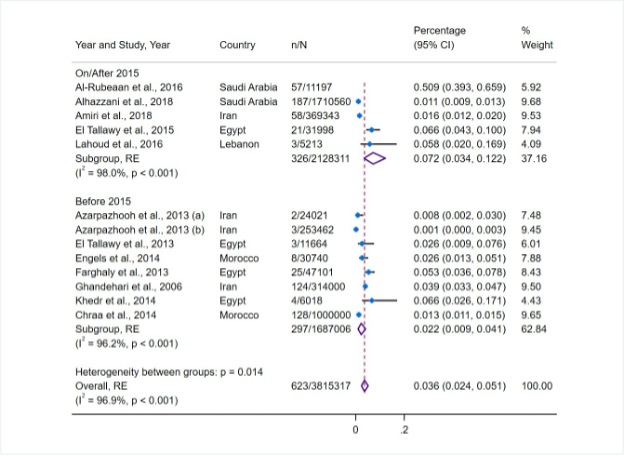

Supplement: S16 Fig — (TIF) [file pgph.0004666.s021.tif]
