# Supplementary material for: Prevalence of stroke in young adults in the Middle East and North Africa Region: A systematic review and meta-analysis
Source: PLOS Glob Public Health. 2025 Oct 6;5(10):e0004666. doi: 10.1371/journal.pgph.0004666 (PMC12500109; doi:10.1371/journal.pgph.0004666)
Supplement: Attachment — Submitted filename: Reviewer reportfinal.docx [file pgph.0004666.s022.docx]

**Reviewer report:**

Prevalence of Stroke in Young Adults in the Middle East and North Africa Region: A Systematic Review and Meta-Analysis

The manuscript had performed a systematic review and meta-analysis that capable to estimate the stroke prevalence in MENA region. It well written and organized.

**Major comments:**

1. Why ages less than 15 and ages greater than 45 are excluded? Which standard age classification is employed to select ages between 15-45 to define young adults?
2. The 14 studies included in the meta –analysis originate from 6 countries i.e. Saudi Arabia, Iran, Egypt, Morocco, Lebanon, and Qatar> Is it logical/epidemiological representative to assume the overall prevalence computed from these applies to the whole	MENA region?

It is notable that the region consists of the following countries……. This region includes Afghanistan, Algeria, Bahrain, Egypt, Iran (Islamic Republic of), Iraq, Jordan, Kuwait, Lebanon, Libya, Morocco, Oman, Palestine, Qatar, Saudi Arabia, Sudan, Syrian Arab Republic (SAR), Tunisia, Turkey, United Arab Emirates (UAE), and Yemen.

3. What is the causative agent for stroke prevalence doubling in the year 2015? It should be discussed and interpreted in the manuscript.
4. Excel extracted data can be included as supplementary data in PLOS as open publication mechanism…
5. In the methodology it is stated that publications in all languages were included but the references did not pin point any additions than English language publications. The region being mainly Arabic speaker…….Is there any attempt to assess Arabic based national or regional publications on the subject?
6. The study is claimed to be a new and Nobel. What values it adds on previous global burden of disease studies that includes the following manuscripts and others not mentioned here?

Shahbandi A, Shobeiri P, Azadnajafabad S, Saeedi Moghaddam S, Sharifnejad Tehrani Y, Ebrahimi N, Rezaei N, Rashidi MM, Ghamari SH, Abbasi-Kangevari M, Koolaji S, Haghshenas R, Rezaei N, Larijani B, Farzadfar F. Burden of stroke in North Africa and Middle East, 1990 to 2019: a systematic analysis for the global burden of disease study 2019. BMC Neurol. 2022 Jul 27;22(1):279. doi: 10.1186/s12883-022-02793-0. PMID: 35896999; PMCID: PMC9327376.

Jaberinezhad, M., Farhoudi, M., Nejadghaderi, S.A. *et al.* The burden of stroke and its attributable risk factors in the Middle East and North Africa region, 1990–2019. *Sci Rep* **12**, 2700 (2022). <https://doi.org/10.1038/s41598-022-06418-x>

Al-Rukn S, Mazya M, Akhtar N, et al. Stroke in the Middle-East and North Africa: A 2-year prospective observational study of intravenous thrombolysis treatment in the region. Results from the SITS-MENA Registry. *International Journal of Stroke*. 2019;15(9):980-987. doi:[10.1177/1747493019874729](https://doi.org/10.1177/1747493019874729)

Soleimani H, Nasrollahizadeh A, Nasrollahizadeh A, Razeghian I, Molaei MM, Hakim D, Nasir K, Al-Kindi S, Hosseini K. Cardiovascular disease burden in the North Africa and Middle East region: an analysis of the global burden of disease study 1990-2021. BMC Cardiovasc Disord. 2024 Dec 19;24(1):712. doi: 10.1186/s12872-024-04390-0. PMID: 39702074;

Mansouri A, Khosravi A, Mehrabani-Zeinabad K, Kopec JA, Adawi KI, Lui M, Rahim HF, Anwar W, Fadhil I, Sulaiman K, Bazargani N. Trends in the burden and determinants of hypertensive heart disease in the Eastern Mediterranean region, 1990–2019: an analysis of the Global Burden of Disease Study 2019. EClinicalMedicine. 2023 Jun 1;60

Vollset SE, Ababneh HS, Abate YH, Abbafati C, Abbasgholizadeh R, Abbasian M, Abbastabar H, Abd Al Magied AH, Abd ElHafeez S, Abdelkader A, Abdelmasseh M. Burden of disease scenarios for 204 countries and territories, 2022–2050: a forecasting analysis for the Global Burden of Disease Study 2021. The Lancet. 2024 May 18;403(10440):2204-56.

**Minor:**

There are variations in the methodologies employed for the 14 studies included in the study with regard to Stroke Definition Diagnostic Criteria Data Collection as shown on Table 1. This in turn might result in different interpretations and it should be emphasized in the discussions and limitations of the study adequately.

Regarding statistical techniques employed it is better if a subject expert on statistics ( Biostatistician) evaluates them.

In conclusion, the study had some unique features worthy of publications in PLOS Global Public health. The authors had performed great piece of work.
