# Supplementary material for: Prevalence of stroke in young adults in the Middle East and North Africa Region: A systematic review and meta-analysis
Source: PLOS Glob Public Health. 2025 Oct 6;5(10):e0004666. doi: 10.1371/journal.pgph.0004666 (PMC12500109; doi:10.1371/journal.pgph.0004666)
Supplement: Attachment — Submitted filename: Response to Reviewers.docx [file pgph.0004666.s024.docx]

**Rebuttal Letter (Response to Reviewers)**

**Manuscript Title:** *Prevalence of Stroke in Young Adults in the Middle East and North Africa Region: A Systematic Review and Meta-Analysis*
**Corresponding Author:** Dr. Tawanda Chivese

Dear Editors and Reviewers,

We would like to thank you for your thoughtful and constructive feedback. We have carefully revised our manuscript in response to the reviewers' comments. Below we provide a point-by-point response, indicating the changes made.

**Reviewer #1 – Comment**

**“This paper is written well and very clear to understand stroke prevalence among young adults. However, I think the introduction can be more specific to show number of stroke rate among young adult from other region for comparison with MENA region.”**

**Response:**
Thank you, we have added this information as shown below:

*“Historically, stroke was perceived as a disease of the elderly, however, it is now increasing in prevalence in young adults globally [8]. In the United States, stroke incidence among adults aged 20–44 has risen from 17 per 100,000 adults in 1993 to 28 per 100,000 adults in 2015 [9].* *In Spain’s Aragon region, the annual incidence of ischemic stroke among adults aged 18–50 was approximately 12.3 per 100,000 between 2005 and 2015 (1). In several Western European populations, the incidence among those under 45 is around 10 per 100,000 (2).*

**Reviewer #2 – Comments**

1. **“Why ages less than 15 and ages greater than 45 are excluded? Which standard age classification is employed to select ages between 15-45 to define young adults?”**

**Response:**

Thank you. The 15–45 age range was selected based on several prior studies defining “young stroke” (e.g., Ji et al., 2013 (3); Nedeltchev et al., 2005 (4); Adams et al.,1986 (5), Bevan et al. 1990 (6), Bukhari et al., 2023 (7)), where 45 is a common upper limit. We chose 15 as the lower age limit because it was the youngest age reported in these foundational studies, particularly in the study by Adams et al. (5). This range allowed us to align with the most frequently used definitions in literature while maintaining consistency across included studies. Moreover, those aged less than 16 are usually children who tend to have a very low risk of stroke unless they have congenital conditions (8), while those aged 45 years and above are usually classified as middle-aged adults. Our classification almost follows the developmental psychology stages by Erikson (9), but the difference is that we used 45 years as our cutoff instead of 40 according to the seen publications on the topic. We have now added this explanation in the Methods section, 2.3. Eligibility criteria to clarify this selection:

*“This age range was selected based on commonly used definitions in stroke literature, where 45 years is often considered the upper limit of "young adult stroke" in global studies (3-6).”*

2. **“The 14 studies included in the meta analysis originate from 6 countries i.e. Saudi Arabia, Iran, Egypt, Morocco, Lebanon, and Qatar> Is it logical/epidemiological representative to assume the overall prevalence computed from these applies to the whole MENA region?
   It is notable that the region consists of the following countries……. This region includes Afghanistan, Algeria, Bahrain, Egypt, Iran (Islamic Republic of), Iraq, Jordan, Kuwait, Lebanon, Libya, Morocco, Oman, Palestine, Qatar, Saudi Arabia, Sudan, Syrian Arab Republic (SAR), Tunisia, Turkey, United Arab Emirates (UAE), and Yemen.”**

**Response:**

We agree this is a limitation. We have now explicitly acknowledged this in the Limitations section, noting that our findings are primarily based on available data from 6 countries and may not generalize to all 20+ MENA countries. We also advocate for expanded data collection in underrepresented nations.

*First, although we aimed to cover the MENA region comprehensively, but all the 14 included studies were from only six countries, namely; Egypt, Iran, Saudi Arabia, Morocco, Qatar, and Lebanon. Thus, the findings may not be generalizable to all MENA countries.*

3. **“What is the causative agent for stroke prevalence doubling in the year 2015? It should be discussed and interpreted in the manuscript.”
   Response:**

Thank you, we chose this year because this was the midpoint of the publication dates, to enable an assessment of any changes in the prevalence of the stroke. The choice was a statistical/ methodological choice, driven by a need to assess trends across the years in the context of very few data points. It is highly unlikely that there was a causative agent in the year but the observed increase in prevalence is likely a reflection of the increasing prevalence over the years. We have expanded the Discussion to clarify that the publication year does not always reflect the data collection year and revised the writing top give a more cautious interpretation.

*“Additionally, the analysis showing an increase in stroke prevalence after 2015 should be interpreted with caution. The grouping was based on publication year rather than year of data collection, which may introduce misclassification.* *The year was selected as it represented the midpoint of the publication timeline, allowing for an evaluation of trends in stroke prevalence over time. While it is unlikely that any specific causative factor emerged in that particular year, the observed doubling in prevalence likely reflects a broader, gradual increase across the studied period.”*

4. **“Excel extracted data can be included as supplementary data in PLOS as open publication mechanism.”
   Response:**

We have compiled the extracted data from all included studies into an Excel file and uploaded it as **Supplementary Table 2**.

5. **“In the methodology it is stated that publications in all languages were included but the references did not pinpoint any additions than English language publications. The region being mainly Arabic speaker…….Is there any attempt to assess Arabic based national or regional publications on the subject?”
   Response:**

Thank you for noting this. Although we did not restrict the search to English studies, we did not identify studies in other languages including Arabic. We have added this statement in the Methods in 2.3. Eligibility criteria.

*“Although no language restrictions were applied during the search, all included studies were published in English or had English abstracts accessible through the databases searched.”*

6. **“The study is claimed to be a new and Nobel. What values it adds on previous global burden of disease studies that includes the following manuscripts and others not mentioned here?”**
   **Response:**

Thank you, we have clarified that this study adds to the existing evidence on the growing threat of stroke in the young. While Global Burden of Disease (GBD) studies such as those by Shahbandi et al. (2022) and Jaberinezhad et al. (2022) offer modeled estimates of stroke burden across all age groups in the MENA region, our study provides an analysis of primary data which are from sources that have not been included in previous sources. This population is underrepresented in most national and global estimates, yet they face rising stroke incidence. Our study is based on direct prevalence data from observational studies rather than modeled projections, and we offer detailed subgroup analyses by stroke subtype (ischemic vs. hemorrhagic), sex, and study setting (hospital- vs. population-based), which are not provided in GBD analyses. A comparative explanation of these differences has now been added to the Discussion section of the manuscript.

*“Unlike prior Global Burden of Disease (GBD) studies such as those by Shahbandi and Jaberinezhad (10, 11), which provide modeled estimates across all ages, our study offers a focused meta-analysis of observational data specific to young adults aged 15–45 years in the MENA region. By using real-world prevalence data, we provide detailed insights on stroke subtypes, sex differences, and study settings—granularity not available in GBD outputs. Additionally, we assess methodological heterogeneity and temporal trends, offering localized, age-specific evidence to inform policy and prevention efforts in the region.”*

7. **“There are variations in the methodologies employed for the 14 studies included in the study with regard to Stroke Definition Diagnostic Criteria Data Collection as shown on Table 1. This in turn might result in different interpretations and it should be emphasized in the discussions and limitations of the study adequately.”
   Response:**

We expanded the Limitations section to discuss how heterogeneity in stroke definition, diagnostic methods, and sampling approaches could impact pooled estimates.

*“Second, there was considerable heterogeneity across included studies in terms of age ranges, study design (cross-sectional, retrospective, or prospective), diagnostic methods (clinical vs. imaging-based diagnosis), screening approach, hospital VS population- based and stroke definitions (WHO vs. local or unspecified criteria). These differences may affect pooled prevalence estimates and limit direct comparability.”*

8. **“Regarding statistical techniques employed it is better if a subject expert on statistics (Biostatistician) evaluates them.”**

**Response:**

Thank you, our study team includes an epidemiologist and a biostatistician, and we have consulted experts in the College too during the design and analysis of the study.

**Reviewer #3 – Comments**

1. **“Can you please expand a bit on your exclusion criteria. For example:
   1. What did you do if a study included people with a larger age range than 15-45 (for example 15-60) did you include it, or exclude it?
   2. What did you do if a study included people with a smaller age range than 14-45 (for example 20-45) did you include it or exclude it?
   3. How did you deal with studies that had data from within MENA and outside MENA?
   4. How did you deal with special populations?
   5. Did you include studies that were based on people with multi-morbidities?”**

**Response:**

Thank you. We updated the Eligibility Criteria section to state that:

- Studies with wider age ranges (e.g., 15–60) were included if disaggregated data for 15–45 could be obtained.
- Studies with narrower ranges (e.g., 20–45) were included.
- Studies with data from both MENA and non-MENA countries were included only if MENA-specific data could be isolated.
- Studies on comorbid or special populations were included if relevant.

*“Studies were eligible if they reported prevalence data (total or subtype: ischemic or hemorrhagic), included age-disaggregated data for the target group (15–45), and provided extractable data specific to MENA populations. We included studies with narrower overlapping age brackets (e.g., 20–45) but excluded those with broader age groups (e.g., 15–60) if relevant age-specific data were not provided. Studies on comorbid or special populations were included if relevant. We excluded commentaries, conference abstracts, narrative reviews, and studies lacking sufficient outcome data or those whose results could not be converted into prevalence estimates.”*

2. **“For risk of bias, many of your readers are probably unfamiliar with Hoy-can you clarify if 10 is the best possible score?”**
   **Response:**

Thank you. We clarified that 10 is the best possible score on the Hoy tool, indicating lowest risk of bias. This is now stated in 2.6. Study risk of bias assessment

*“This tool evaluates 10 domains covering external and internal validity. Each domain was rated as “Yes” (low risk) or “No” (high risk), and total scores ranged from 0 (high risk of bias) to 10 (low risk). A score of 8 or more was interpreted as low risk, consistent with published guidelines.”*

3. **“I see that you included several studies that were hospital based. Hospital based studies can be problematic in conducting prevalence studies as it can be challenging to understand the denominator that the population is coming from-use of hospital based studies generally inflates prevalence estimates. I would like to see some mention of how you dealt with this, and how you estimated the denominators.”**
   **Response:**

We acknowledge that hospital-based studies are problematic in estimating prevalence due to a lack of screened denominators. However, we included them because there is a general lack of studies and for completeness of reporting the available data. We have carried out a subgroup analysis of the population/cross-sectional data compared to the hospital-based studies in the results section. We have added this to the discussion.

*“In particular, hospital-based studies may overestimate or under-estimate stroke prevalence due to selection bias and unclear or unrepresentative denominators. Although we conducted subgroup analysis by study setting (population- vs. hospital-based), some uncertainty remains.* *However, given the overall scarcity of data in the region, these studies were included to ensure a more comprehensive and representative synthesis of the available evidence.”*

4. **“I was a bit surprised to see that all of your studies were a low risk of bias. You may want to go through those again. I have done a fair number of systematic reviews and have never done one where every single study was low risk of bias.”**

**Response:**

Thank you, we have revised the risk of bias. We changed the scoring in Supplementary Table 1 and updated the paragraph 3.3. Risk of bias in studies as follows:

*“The 14 included studies demonstrated a wide range in methodological quality. Eight studies (12-19) scored 8 out of 10, indicating a low risk of bias and strong adherence to internal and external validity. The study by Al-Rubeaan (20) scored 7, while by Azarpazhooh (21) scored 6, and four studies (22-25) received scores of 4, reflecting a higher risk of bias. The most frequent sources of bias were related to non-representative sampling frames, lack of national representativeness in the target population, and insufficient information on the reliability or validity of the measurement instruments used to assess stroke prevalence. These limitations were particularly evident in studies that relied on hospital-based convenience samples or lacked methodological transparency. Nevertheless, most studies used consistent case definitions and data collection procedures, supporting the overall robustness of the findings. Detailed item-by-item scoring is provided in Supplementary Table 1.”*

5. **“At the beginning of section 3.4.1 you state that 12 studies were included for meta-analysis, but do not explain why some were excluded. In your results you need to have a section were you explain this. You may also want to include this in your prisma diagram.”**

**And**

**“You start the discussion by saying that this is a systematic review and meta-analysis of 14 studies, but only 12 studies are included in the meta-analysis.”**

**Response:**

Thank you for this helpful observation. We have clarified in the Results section that two of the fourteen included studies did not report the overall prevalence of stroke in young adults and therefore were excluded from the general pooled prevalence analysis. However, both studies reported segregated prevalence data for ischemic and hemorrhagic stroke subtypes, so they were included in the corresponding subtype analyses. We have also updated the PRISMA diagram to reflect this distinction and added a note to ensure this is clearly communicated to readers.

Under Figure 1: PRISMA Flow Diagram of Study Selection Process

*“*Two studies excluded from the overall prevalence meta-analysis due to missing total prevalence data but included in subtype analyses.”*

In 3.4.1. Stroke prevalence

*“**Two additional studies (22, 25) were excluded from this analysis due to missing total prevalence data but were included in the ischemic and hemorrhagic subtype analyses.”*

6. **“In section 3.4.4 when you discuss trends over time, it would be helpful to also discuss the number of studies in each time frame, the number of individuals counted, and the number of events. Given the small number of studies in the two time frames, and the heterogeneity I do not think this finding warrants as much emphasis as you are putting on it. Four of the six post 2015 studies were in either 2015 or 2016, and seven of the eight pre 2015 studies were from 2013 or 2014. So basically what your findings are saying is that this change occurred between 2013 and 2016, which is both unrealistic, and not how you are presenting the findings. Additionally this is just based on publication date, so to do this properly you would want to look at when the strokes were actually occurring.”**
   **Response:**

Thank you for your thoughtful comment. In response, we have made the following revisions to improve clarity and address the concerns raised:

1. We have added the requested data on the number of studies, individuals assessed, and stroke events in each time frame to the Results section 3.4.4. Trends in the prevalence of stroke in the young over time:

*“Among the 8 studies (12, 16-19, 21, 24, 25) conducted before 2015, a total of 1,686,006 individuals were assessed, with 352 reported stroke events. In contrast, the 6 studies (13-15, 20, 22, 23) conducted in 2015 or later included a larger combined sample of 2,128,311 individuals and reported 326 stroke events.”*

2. We have revised the Discussion section to clearly acknowledge that the publication year does not necessarily reflect the data collection period, and was added to the Limitations section:

*“Additionally, the analysis showing an increase in stroke prevalence after 2015 should be interpreted with caution. The grouping was based on publication year rather than year of data collection, which may introduce misclassification”*

3. We have softened the language throughout the relevant sections to ensure a more tentative and cautious interpretation of the observed trends. The emphasis on changes over time has been reduced:

In the Results section in 3.4.4. Trends in the prevalence of stroke in the young over time

*“The p for interaction (p=0.014) indicates a statistically significant difference in pooled prevalence estimates between the two periods; however, this should be interpreted with caution due to the small number of studies and high heterogeneity.”*

Instead of *“The p for interaction (p=0.014) suggested strong evidence of changes in prevalence by time. The analysis results suggested an increase in prevalence estimates over time during the periods analyzed.”*

In the Discussion

*“Although stroke prevalence in the MENA region remains at the lower than other global estimates, our analysis suggests a rising prevalence over time, with studies conducted on or after 2015 reporting a higher pooled prevalence (0.072%) compared to those before 2015 (0.022%). These findings suggest an increase in the numbers of affected young adults over a short period of time. The findings underscore the need to address the risk factors for stroke, enhanced screening, and consider allocations of more resources for the treatment of stroke and rehabilitation in the region in young adults. However, this should not be interpreted as a confirmed increase in stroke prevalence over time. Additional high-quality, population-based studies with clearly defined time frames are necessary to accurately assess temporal changes.”*

Instead of *“**Although stroke prevalence in the MENA region remains at the lower end, our analysis suggests a rising trend over time, with studies conducted on or after 2015 reporting a significantly higher pooled prevalence (0.072%) compared to those before 2015 (0.022%). These findings suggest a rapid increase in the numbers of affected young adults over a short period of time. The findings underscore the need to address the risk factors for stroke, enhanced screening, and consider allocations of more resources for the treatment of stroke and rehabilitation in the region in young adults.”*

7. **“In the results you mention that smaller studies tended to have greater prevalences. There is a fair bit of literature on how smaller size studies tend to overestimate effect sizes. You may want to mention this in the discussion.”**
   **Response:**

We added a sentence in the *Discussion* referencing literature that small studies often overestimate effects and discussed this as a possible contributor to heterogeneity.

*“Finally, smaller studies appeared to report higher prevalence estimates (26, 27). This may reflect publication bias or small study effects, a known trend in meta-analyses where small studies tend to overestimate effect sizes. Although we used funnel and Doi plots to detect this, and conducted sensitivity analyses, we recommend cautious interpretation of these estimates.”*

8. **“I would expect to see some discussion about how your results compare to the results in the GBD-you can get estimates for 15-45 by individual country or region and by year, so it would be interesting to see how similar or different your results are.”**
   **Response:**

This comparison is now included in *Discussion*, with GBD estimates where applicable.

*“Compared to GBD estimates, our findings suggest a lower absolute prevalence in young adults (15–45 years). GBD 2021 data indicate that 14.8% of all stroke cases occur in the 15–49 age group globally (28), and reports age-standardized stroke prevalence across all ages in the MENA region was approximately 607 per 100,000 (11). In our study, the pooled prevalence in 15–45-year-olds was 36 per 100,000—substantially lower than the GBD estimate, but it should be noted that our meta-analytic estimate is not comparable to an age standardized estimate. The age standardized prevalence from the GBD is for comparative purposes and is highly dependent on the standardizing population used. Additionally, the GBD suggests that the age standardized prevalence of stroke in the MENA went down from 744 per 100,000 in 1990 to 607 per 100,000 in 2021, a trend that could not be assessed in the current meta-analysis because of a lack of data sources. Our findings, together with the GBD estimates highlight a need for more robust data on stroke in young people in the MENA region.”*

9. **“You may want to expand your limitations section and be a bit more specific about sources of heterogeneity. There are different age ranges, sample sizes etc.”**
   **Response:**

The Limitations section has been expanded to explicitly mention heterogeneity sources.

*“Second, there was considerable heterogeneity across included studies in terms of age ranges, study design (cross-sectional, retrospective, or prospective), diagnostic methods (clinical vs. imaging-based diagnosis), screening approach, hospital VS population- based and stroke definitions (WHO vs. local or unspecified criteria). These differences may affect pooled prevalence estimates and limit direct comparability.”*

10. **“The final paragraph of your discussion and as well as much of your conclusion focusses on the rising prevalence of stroke, but given the small sample size and very large heterogeneity, I am not sure you can make this strong of a statement based on your results.”**

**Response:**

Thank you we have revised this section to be more tentative and to emphasize the need for more rigorous and good quality data:

In Conclusion:

*“This systematic review and meta-analysis revealed an overall stroke prevalence of 0.036% among young adults in the MENA region, with ischemic stroke being the predominant subtype and significant heterogeneity across countries. Gender-specific analyses showed comparable prevalence in males and females, although certain countries exhibited higher rates among women. Our analysis also suggested that the prevalence of stroke in young adults in the region may be increasing, although the data sources were few and of mostly low quality. More robust, standardized, and representative epidemiological studies are needed to accurately assess the burden of stroke and its trends among young adults in the MENA region.”*

Instead of *“more high-quality primary studies are needed to provide a clearer estimate of prevalence and trends in the prevalence of stroke in young adults in the MENA region.”*

**Note regarding figure files:**
All figure files have been processed using the Preflight Analysis and Conversion Engine (PACE) to ensure compliance with PLOS formatting requirements. The final, PACE-validated versions have been uploaded with this revised submission.

We hope these revisions address your concerns and improve the clarity and scientific value of our manuscript. Thank you again for your valuable feedback and the opportunity to revise our work.

Sincerely,
Dr. Tawanda Chivese and Salma Hegazi (on behalf of all authors)

**References**

1.	Tejada Meza H, Artal Roy J, Pérez Lázaro C, Bestué Cardiel M, Alberti González O, Tejero Juste C, et al. Epidemiology and characteristics of ischaemic stroke in young adults in Aragon. Neurologia (Engl Ed). 2022;37(6):434-40.

2.	Gandolfo C, Conti M. Stroke in young adults: epidemiology. Neurological Sciences. 2003;24(1):s1-s3.

3.	Ji R, Schwamm LH, Pervez MA, Singhal AB. Ischemic Stroke and Transient Ischemic Attack in Young Adults: Risk Factors, Diagnostic Yield, Neuroimaging, and Thrombolysis. JAMA Neurology. 2013;70(1):51-7.

4.	Nedeltchev K, der Maur TA, Georgiadis D, Arnold M, Caso V, Mattle HP, et al. Ischaemic stroke in young adults: predictors of outcome and recurrence. Journal of Neurology, Neurosurgery &amp;amp; Psychiatry. 2005;76(2):191.

5.	Adams HP, Jr., Butler MJ, Biller J, Toffol GJ. Nonhemorrhagic Cerebral Infarction in Young Adults. Archives of Neurology. 1986;43(8):793-6.

6.	Bevan H, Sharma K, Bradley W. Stroke in young adults. Stroke. 1990;21(3):382-6.

7.	Bukhari S, Yaghi S, Bashir Z. Stroke in Young Adults. Journal of Clinical Medicine [Internet]. 2023; 12(15).

8.	Ferriero DM, Fullerton HJ, Bernard TJ, Billinghurst L, Daniels SR, DeBaun MR, et al. Management of Stroke in Neonates and Children: A Scientific Statement From the American Heart Association/American Stroke Association. Stroke. 2019;50(3):e51-e96.

9.	Erikson EH. Childhood and Society. 2nd ed. New York: W.W. Norton & Company; 1963.

10.	Streletz L, A M, Gad H, Abbasi S, Dm D, Akhtar N, et al. Epidemiology of Stroke in the MENA Region: A Systematic Review. 2017.

11.	Jaberinezhad M, Farhoudi M, Nejadghaderi SA, Alizadeh M, Sullman MJM, Carson-Chahhoud K, et al. The burden of stroke and its attributable risk factors in the Middle East and North Africa region, 1990–2019. Scientific Reports. 2022;12(1):2700.

12.	Khedr EM, Fawi G, Abdela M, Mohammed TA, Ahmed MA, El-Fetoh NA, Zaki AF. Prevalence of Ischemic and Hemorrhagic Strokes in Qena Governorate, Egypt: Community-based Study. Journal of Stroke and Cerebrovascular Diseases. 2014;23(7):1843-8.

13.	Lahoud N, Salameh P, Saleh N, Hosseini H. Prevalence of Lebanese stroke survivors: A comparative pilot study. J Epidemiol Glob Health. 2016;6(3):169-76.

14.	Amiri A, Kapral MK, Thrift AG, Sposato LA, Saber H, Behrouz R, et al. The Incidence and Characteristics of Stroke in Urban-Dwelling Iranian Women. Journal of Stroke and Cerebrovascular Diseases. 2018;27(3):547-54.

15.	El Tallawy HN, Farghaly WM, Badry R, Hamdy NA, Shehata GA, Rageh TA, et al. Epidemiology and clinical presentation of stroke in Upper Egypt (desert area). Neuropsychiatr Dis Treat. 2015;11:2177-83.

16.	El-Tallawy HN, Farghaly WM, Shehata GA, Abdel-Hakeem NM, Rageh TA, Badry R, Kandil MR. Epidemiology of non-fatal cerebrovascular stroke and transient ischemic attacks in Al Quseir, Egypt. Clin Interv Aging. 2013;8:1547-51.

17.	Engels T, Baglione Q, Audibert M, Viallefont A, Mourji F, El Alaoui Faris M, for the GSG. Socioeconomic Status and Stroke Prevalence in Morocco: Results from the Rabat-Casablanca Study. PLOS ONE. 2014;9(2):e89271.

18.	Farghaly WM, El-Tallawy HN, Shehata GA, Rageh TA, Abdel-Hakeem NM, Elhamed MA, et al. Epidemiology of nonfatal stroke and transient ischemic attack in Al-Kharga District, New Valley, Egypt. Neuropsychiatr Dis Treat. 2013;9:1785-90.

19.	Ghandehari K, Moud ZI. Incidence and etiology of ischemic stroke in Persian young adults. Acta Neurol Scand. 2006;113(2):121-4.

20.	Al-Rubeaan K, Al-Hussain F, Youssef AM, Subhani SN, Al-Sharqawi AH, Ibrahim HM. Ischemic Stroke and Its Risk Factors in a Registry-Based Large Cross-Sectional Diabetic Cohort in a Country Facing a Diabetes Epidemic. J Diabetes Res. 2016;2016:4132589.

21.	Azarpazhooh MR, Shahripour RB, Kapral MK, Mokhber N, Shoeibi A, Farzadfard MT, et al. Incidence of first ever stroke during Hajj ceremony. BMC Neurology. 2013;13(1):193.

22.	Abujaber AA, Imam Y, Nashwan A, Own AM, Akhtar N. Stroke in Qatar: a decade of insights from a national registry. Neurological Research. 2024;46(10):893-906.

23.	Alhazzani AA, Mahfouz AA, Abolyazid AY, Awadalla NJ, Aftab R, Faraheen A, Khalil SN. Study of Stroke Incidence in the Aseer Region, Southwestern Saudi Arabia. Int J Environ Res Public Health. 2018;15(2).

24.	Chraa M, Louhab N, Kissani N. Stroke in young adults: about 128 cases. Pan Afr Med J. 2014;17:37.

25.	Khan FY, Yasin M, Abu-Khattab M, El Hiday AH, Errayes M, Lotf AK, et al. Stroke in Qatar: A First Prospective Hospital-based Study of Acute Stroke. Journal of Stroke and Cerebrovascular Diseases. 2008;17(2):69-78.

26.	Zhang Z, Xu X, Ni H. Small studies may overestimate the effect sizes in critical care meta-analyses: a meta-epidemiological study. Crit Care. 2013;17(1):R2.

27.	Nüesch E, Trelle S, Reichenbach S, Rutjes AW, Tschannen B, Altman DG, et al. Small study effects in meta-analyses of osteoarthritis trials: meta-epidemiological study. Bmj. 2010;341:c3515.

28.	Zhang M, Long Z, Liu P, Qin Q, Yuan H, Cao Y, et al. Global Burden and Risk Factors of Stroke in Young Adults, 1990 to 2021: A Systematic Analysis of the Global Burden of Disease Study 2021. Journal of the American Heart Association. 2025;14(10):e039387.
